# The effect of bilingualism on executive functions when languages are similar: a comparison between Hungarian–Serbian and Slovak–Serbian young adult bilinguals

**DOI:** 10.3758/s13421-022-01345-8

**Published:** 2022-07-29

**Authors:** Alexandra Perovic, Dušica Filipović Đurđević, Sabina Halupka-Rešetar

**Affiliations:** 1 Division of Psychology and Language Sciences, University College London, London, UK; 2 Department of Psychology, University of Belgrade, Faculty of Philosophy, Belgrade, Serbia; 3 Laboratory for Experimental Psychology, University of Belgrade, Faculty of Philosophy, Belgrade, Serbia; 4 Laboratory for Experimental Psychology, University of Novi Sad, Faculty of Philosophy, Novi Sad, Serbia; 5 Department of English Studies, University of Novi Sad, Faculty of Philosophy, Novi Sad, Serbia

**Keywords:** Bilingualism, Executive functions, CANTAB, Language similarity

## Abstract

Among the factors argued to contribute to a bilingual advantage in executive function (EF), the combination of languages spoken by the bilingual is often overlooked. In this study, we explored the role of language similarity on memory and EF task by comparing performance of three groups of young adults—Hungarian–Serbian and Slovak–Serbian early balanced bilinguals, and Serbian-speaking monolinguals. Slovak is typologically related to Serbian, which are both Slavic, in contrast to Hungarian, which is Finno–Ugric. On the computerized tasks from the CANTAB battery (CANTAB Cognition, 2016, www.cantab.com), differences between monolinguals and bilinguals emerged on the EF tasks: Stockings of Cambridge (SOC) and Attentional Set Shifting (AST), but not the memory tasks: Delayed Matching to Sample (DMS), Paired Associate Learning (PAL), Spatial Working Memory (SWM). Both Hungarian–Serbian and Slovak–Serbian bilinguals outperformed the monolinguals on the more difficult SOC tasks, solved using more than a minimally required number of moves. This is in line with reports that bilinguals perform better under more complex conditions that require more monitoring and switching. However, bilinguals speaking Hungarian and Serbian spent less time preparing to execute the simpler SOC tasks, which can be solved in a minimum of two or three moves; they also exhibited reduced local switching cost and were faster overall on AST than both the Slovak–Serbian bilinguals and Serbian monolinguals. The advantageous performance of speakers of the typologically unrelated languages in our study suggests that these bilinguals may have more efficient attention switching and inhibition systems than bilinguals who speak typologically similar languages.

**Supplementary Information:**

The online version contains supplementary material available at 10.3758/s13421-022-01345-8.

A large body of literature reports advantages for bilingual speakers over monolinguals in tasks that tap into different domains of cognitive abilities (Bialystok et al., 2005; see Grundy, 2020; Valian, 2015, for recent reviews). Fluent bilinguals demonstrate a degree of activation of both languages and some interaction between them, even in contexts in which only one of the languages is being used. Bilinguals are thus faced with an attention problem caused by the need to select the appropriate language for the context, which may make other cognitive processes more effortful. However, at the same time, the need to resolve competition and to focus attention may result in an advantage in executive functioning (from here on, EF), which includes the set of cognitive skills that exploit limited cognitive resources for functions such as inhibition, attention switching, and working memory (Akhtar & Menjivar, 2012; Miyake et al., 2000). The results of existing research suggest that bilinguals sometimes display an advantage in inhibition and selection, in switching and sustaining attention, and in working memory as well as representation and retrieval (see, i.e., Grundy, 2020; Monnier et al., 2021). This suggests that bilinguals can adapt easily to ongoing changes and that they can process information efficiently and adaptively, showing signs of ‘mental flexibility’ (Bialystok et al., 2012).

However, a growing number of reports have questioned the existence of demonstrable differences in the cognitive performance of bilinguals versus monolinguals (see a recent meta-analysis by Lehtonen et al., 2018, and reviews by, e.g., Paap, 2019; Paap et al., 2015). Concerns of “positive bias” in the literature have also been raised, where it has been argued that the publication of studies on bilingualism and executive control is contingent on reporting positive results only (de Bruin et al., 2015). Importantly, results showing a lack of cognitive advantage in bilingualism seem more frequent in studies conducted with young adults, as opposed to children or older adults (e.g., Bialystok et al., 2005; Colzato et al., 2008; Costa et al., 2008; Grundy & Timmer, 2017; Lehtonen et al., 2018). This pattern is argued to be a consequence of young adults reaching a peak performance in terms of their ability to exercise executive control (Bialystok, 2006; Bialystok et al., 2012; Costa et al., 2009). While ceiling effects are common in the performance of both monolingual and bilingual young adults (Grundy, 2020), bilingual young adults have been found to outperform their monolingual peers only (or especially) under more complex conditions, which require an extra level of monitoring and switching (Bialystok, 2006; Costa et al., 2009; Hernández et al., 2010). This result has been interpreted as evidence that young adult bilinguals have a more efficient monitoring system for conflict resolution (Bialystok et al., 2012). Other studies have argued that it is the heterogeneity of the bilinguals’ linguistic backgrounds that have contributed to the controversies surrounding bilinguals’ advantage in EF, citing several factors: the varying definitions of bilingualism (simultaneous vs. sequential), language proficiency (less vs. more proficient), the context in which languages are used (single vs. dual language context, e.g., workplace or home, with same or different speakers), age of L2 acquisition (early vs. late), differences in participants’ backgrounds (i.e. education, socioeconomic status, immigrant and cultural background), to task characteristics (verbal vs. nonverbal) as well as sample size effects and variability in power (e.g., Chamorro & Janke, 2020; Green & Abutalebi, 2013; Grundy, 2020; Leivada et al., 2021; Valian, 2015).

However, among the factors argued to contribute to a bilingual advantage in EF in adults, one issue that is often overlooked is the combination of languages spoken by the bilingual. If the benefits of bilingualism are at least partly explained by the joint activation of two languages, the question arises as to whether language typology plays a role—that is, whether bilingualism in typologically related languages uses more or less attentional control to maintain separation, than bilingualism in unrelated languages.

It has been argued that the structural and lexical similarity of the languages spoken by a bilingual creates more interference and therefore requires more control to discriminate between them: This may lead to more enhanced cognitive control mechanisms in speakers of typologically related languages (Bialystok, 2017). However, it is also plausible that bilingualism in more distant or typologically unrelated languages requires more switching and monitoring, which may lead to greater enhancement of executive functions. Language typology is rarely controlled for in studies of bilingualism and EF: studies that include the same language combination for all participants are less frequent (e.g., Antón et al., 2019; Catalan-Spanish, Costa et al., 2008; Basque-Spanish) than studies which recruit participants speaking a variety of language pairs (e.g., 19 languages spoken by 31 bilinguals in Wiseheart et al., 2016). This makes it difficult to determine whether it is a specific language or the relation between the two languages involved that affects the results obtained (this may be further complicated by sociolinguistic factors such as perceived prestige of a language). The literature focusing specifically on the role of linguistic proximity in bilinguals and EF is sparse and the findings are inconclusive. In a recent meta-analysis, Lehtonen et al. (2018) report a small advantage in monitoring (but not other EF domains) in speakers of related languages, Catalan–Spanish, but also of unrelated languages, English–Chinese, over English–Spanish bilinguals. They found no evidence that the language pairs of bilinguals in the reviewed studies influenced the outcomes for other EF domains (e.g., inhibition). Again, it seems difficult to disentangle the influence of methodological factors such as the type of task employed and variables related to bilingualism (e.g., age of acquisition and language use). A study on Spanish–English, French–English, and Chinese–English bilingual children (Barac & Bialystok, 2012) pointed to advantages for bilinguals on nonverbal EF tasks independently of language similarity, though language similarity played a role in children’s performance on non-EF language tasks. The typological relatedness of languages in the study was not clear however: English, a Germanic language, and Spanish, a Romance language, were considered to be close typologically, despite belonging to distinct language families. In a study investigating whether bilingualism modulates auditory selective attention in early young adult bilinguals, speakers of English and another Germanic language, Dutch, showed a better performance on a language-based task (dichotic listening) than the bilingual speakers of English and a Romance language, Spanish (Olguin et al., 2019). On nonverbal EF tasks, a recent study reported that a smaller typological distance was associated with better performance in young adult late bilinguals (Khodos et al., 2021). A smaller typological distance was defined as the combination of English and another Germanic language, and a larger typological distance for a combination of English and a non-Germanic language - a somewhat coarse dichotomy considering that the participants in this study spoke a wide range of languages: from Germanic, Romance and Slavic in the Indo-European language family, to Chinese and Burman in the Sino-Tibetan language family, Turkic in the Altaic language family, to Benue-Congo in the Niger-Congo language family. Other factors relevant to participants’ linguistic experience in this study played an even more important role however: onset age of active bilingualism, and especially the interactional context of language use. Bilingual participants were exposed to English as an L2 around the age of 9, but did not start actively using English until adulthood, upon arrival to Australia, around the of age 21. However, the key finding that participants who used their languages in dual language contexts (in the same interactional context, but with different speakers) performed better than single language context participants, was confounded by the correlation of language proficiency and language use, with the dual context speakers being more proficient in English. While the role of language typology may be difficult to tease apart from the effects of other variables*,* the results of this study indicate the importance of a range of factors related to bilingual language experience, amongst which the role of linguistic similarity has thus far received inadequate attention.

To shed more light on the repercussions of language typology on the EF skills of bilingual adults’ language processing, the current study investigates the performance of two groups of young bilingual adults and one group of young monolingual adults, on a series of nonverbal EF and memory tasks, while controlling for a number of factors relevant to participants’ language experience. Our bilingual participants are speakers of two typologically similar languages, namely Slovak and Serbian (both are Slavic, inflectional languages), and speakers of two typologically distant languages, Serbian and Hungarian (which is a Finno-Ugric, agglutinative language), who will be compared with monolingual Serbian speakers. In line with suggestions from recent reviews of the literature (e.g., de Bruin, 2019), we control for factors which may contribute to performance on EF tasks by using a homogenous population of young balanced bilingual adults, matched on education and immigration status but also on the variables such as language proficiency, age of acquisition, and language use.

## Setting the scene: characteristics of Serbian, Slovak, and Hungarian; Bilingualism in Vojvodina, Serbia

Serbian is a South Slavic language, highly inflected, with a rich morphology on nouns, verbs, adjectives and pronouns that is accompanied by a relatively free word order. Slovak, a West Slavic language, is similar to Serbian, in that it also has a complex system of morphology and a relatively flexible word order. Hungarian, a Finno-Ugric language belonging to Uralic languages, is very different to Slavic languages in terms of morphology, being an agglutinative language. Like Serbian and Slovak, its basic word order is SVO, but Hungarian is a topic-prominent language, which means that word order is only partly determined by the syntax.

Serbian is the official language in Serbia and the majority language in Vojvodina, an autonomous province in northern Serbia with a unique history of ethnic heterogeneity and multilingualism. Over 80% of Vojvodina’s municipalities are ethnically heterogeneous, but Hungarians and Slovaks are the two largest minority groups. The specific political, social, cultural and educational context that supports Vojvodinian minority languages allows bilingual speakers in this northern Serbian province to become successful bilinguals, who are pragmatically and communicatively competent users of both their languages.

## Experiment: methods

### Participants

Our participants were all non–immigrant nationals of Serbia, residing in Novi Sad, the capital of the Serbian province of Vojvodina, at the time of testing. All bilingual^1^ participants and most of the monolinguals were born and raised in Vojvodina, while a small number of monolinguals came from Serbia proper. The final sample consisted of 71 Serbian monolinguals (61 females) and 40 bilinguals (33 females): 21 Hungarian–Serbian bilinguals and 19 Slovak–Serbian bilinguals,^2^ all students at the University of Novi Sad at the time of testing.^3^ The monolingual participants were psychology majors while the bilinguals studied a range of subjects, from engineering to medicine. Bilingual participants (*M*_age_ = 20.95 years, range: 18–28 years) were slightly older than the monolinguals (*M*_age_ = 19.37 years, range: 18–33 years), *t*(66.266) = 3.6199, *p* = .0006, but the bilingual groups did not differ in age, *t*(34.37) = .38284, *p* = .7042 (mean age of Hungarian–Serbian bilinguals = 20.81 years, range: 18–26 years, and the mean age of Slovak–Serbian bilinguals = 21.11 years, range: 19–28).

For our monolingual group, the recruitment criteria required participants to have lived all their lives in Vojvodina/Serbia, and to have been schooled in Serbian, without being exposed to another home language (foreign languages learnt at school were not taken to be equivalent to home languages).^4^ For the bilingual group, our criteria were created to target members of the Slovak and Hungarian ethnic minorities who were early and balanced bilinguals: They must have lived all their lives in Vojvodina, have been exposed to both of their languages at home before or around the age of 3, and used both their languages in everyday life. Following de Bruin (2019), we put extra effort into the description of the language experience of our two groups of bilingual participants, relying on an instrument that allowed us to zoom in on crucial variables: age of acquisition, language proficiency, and language use. The self-rating Bilingual Language Profile (BLP) scale (Birdsong et al., 2012) probes into the following areas of language dominance, as addressed in the four components of the BLP for each participant’s language: (1) Language History (age of acquisition; years of language learning in different contexts, including educational and social contexts; overall use of each language), (2) Language Use (in an average week for each language, in different contexts, including educational and social contexts), (3) Language Proficiency (a self-assessment ranking of the four language skills in each of the participant’s languages), and (4) Attitude to each language. Table 1 gives the bilingual participants’ BLP mean scores for both their languages, as well as their Global Language Score (GLS; maximum possible score 218), the sum of scores for the four equally weighted components.^5^ Independent *t* tests showed no significant difference between the two bilingual groups on any of the four BLP dimensions, either with respect to the minority language or to the majority language. Our two bilingual groups did not differ in terms of language use or proficiency, nor language exposure: Both groups acquired their home languages from birth or before the age of 3 (1.7 years for Slovak bilinguals, and 2.6 years for Hungarian bilinguals).

**Table 1:** Average scores on the Bilingual Language Profile for the two groups of bilingual participants

Bilingual group	Language	GLS	Exposure	Use	Proficiency	Attitude
Hungarian–Serbian (*N* = 21)	Hungarian	169.27	41.08	27.71	52.10	51.02
	Serbian	136.80	31.24	20.14	47.02	40.32
Slovak–Serbian (*N* = 19)	Slovak	174.36	42.01	33.40	49.58	51.13
	Serbian	145.12	37.42	16.96	50.42	41.22

*GLS*: Global Language Score

### Procedure

The study was approved by the Ethics Boards of the University of Novi Sad and University College London. The current experiments were carried out as a part of a larger study on language and cognition in typically and atypically developing Serbian speakers. Written informed consent was obtained from all the participants. Participants were tested on a series of computerized nonverbal tasks that tap into memory and EF from the Cambridge Neuropsychological Test Automated Battery (CANTAB; Cambridge Cognition, 2016).^6^ The use of a uniform, culturally unbiased battery (Demeyere et al., 2021), replicable across populations, allowed us to establish baseline results of cognitive functioning in young healthy adults, both monolingual and bilingual, who are speakers of languages underrepresented in the research literature.

The testing was carried out during 45–60-minute-long sessions in a soundproof room in the Laboratory for Experimental Psychology at the Faculty of Philosophy, University of Novi Sad. Bilingual participants were tested on the complete battery of CANTAB tasks (explanations of tasks to follow) in the course of two sessions. In the first session, they were administered the language background questionnaire and two of the CANTAB tasks: Delayed Matching to Sample (DSM) and Stockings of Cambridge (SOC). The remaining tasks were administered in the second session: Paired Associates Learning (PAL), Spatial Working Memory (SWM), and Attention Shifting Task (AST). For organizational reasons we were not able to administer all the tests to each monolingual speaker but in line with the standard literature (Harris, 2013; Johnson & Christensen, 2019), we randomly assigned participants from the highly homogenous, much larger group of monolinguals into one of the two groups tested on the following parts of the test battery: DSM and SOC versus PAL, SWM, and AST. Thus, 39 monolingual participants were tested on DMS and SOC, and 32 monolingual participants were tested on PAL, SWM, and AST. In the analyses to be reported, we treat these two groups of monolingual participants as one homogenous group whose performance is compared with that of the two smaller groups of bilingual participants. The participants did not receive remuneration for taking part in the study.

### Materials

**Delayed Matching to Sample (DMS)** taps into immediate and delayed visual matching. At each trial, the participant is briefly presented with a sample visual pattern at the top of the screen and instructed to indicate from the four choice patterns presented at the bottom of the screen the one which is identical to the sample. The four choice patterns are presented either while the target is still present on the screen, immediately following the target presentation, or after a delay of 4 or 12 seconds. In addition to testing perceptual abilities, the delayed conditions also test the capability of maintaining the visual information in memory. The measure of performance is *the proportion of choosing the correct pattern on the first attempt* in each of the delay conditions.

**Paired Associates Learning (PAL)** tests episodic memory—namely, the ability to bind various information into a single episode. In this case, the binding is to be performed on visual and spatial information, as the identity of the visual object needs to be paired with its location. On each trial, a certain number of boxes are presented on the screen, which open one at a time and reveal whether they contain a hidden visual pattern. The task is to remember the location of each hidden pattern and to tap the box that reveals the pattern upon presentation. The successful attempt leads to the next stage which contains more patterns and/or more boxes, whereas the failed attempt is followed by a repeated presentation. The cycle is repeated either until the task is accomplished, or the test is aborted. The core performance measure is *the first trial memory score* (i.e., the number of correct responses on the first attempt).

**Spatial Working Memory (SWM)** addresses two working memory domains, the short-term storage and the updating of information (Owen et al., 1996). It therefore taps into working memory capacity, but also into the executive functioning. The participants are presented with multiple boxes which they must tap in order to find a hidden token. The task is to collect a predetermined number of tokens. However, one should not return to (re-tap) the box from which a token has already been collected. There are several stages of increasing difficulty, as they contain an increasing number of boxes/tokens. The crucial measure is *the number of between errors* (i.e., the number of revisits to the already cleared boxes). Given the self-ordered nature of the task, an additional measure of executive functioning can be calculated. This *strategy* measure represents the number of specific boxes used to begin the search for a token. Strategy is calculated as the number of different boxes used to start the new search, and the higher number reflects the lack of strategy (i.e., the worse achievement).

**Stockings of Cambridge (SOC)**, based on the Tower of London task (Robbins et al., 1998), is a typical test of the frontal lobe executive function, namely planning. Planning has been argued to involve multiple EF skills such as inhibition, updating and switching (Miyake & Friedman, 2012). However, though SOC is generally considered to be tapping into planning abilities, some argue that it is in fact the inhibition abilities that are called upon here: in order to solve the task, participants need to successfully *inhibit* inappropriate moves and select the appropriate ones (Carder et al., 2004; Miyake et al., 2000).

In this task, participants are presented with two visual displays, each consisting of three coloured balls hanging in three stockings. The upper display brings a pattern that the participant must reproduce at the bottom display following certain rules. The problems vary in difficulty (i.e., in the number of moves needed to achieve the desired pattern). The measure typically obtained from this task is *the number of problems solved in minimum moves*. Additionally, *the initial thinking time* can be assessed (i.e., the time taken to plan the moves prior to moving the first ball). The CANTAB manual suggests that the initial thinking time measure may not be sensitive enough to assess high functioning populations such as healthy adults (Cambridge Cognition, 2016). With this in mind, we constructed an additional measure that is calculated as *the initial thinking time for the problems that were solved in a nonminimum number of moves* (i.e., that participants were able to solve, but that took them more effort). We believed that this measure would be more sensitive to variation amongst the student population known for ceiling performance on similar tasks (Grundy, 2020).

**Attention Shifting Task (AST)** taps the executive function of shifting attention in the presence of visual cues. Each trial consists of an arrow presented on one side of the screen and the written reminder of the current task, which is presented on the top of the screen. The task for the participant is either to indicate the side of the screen on which the arrow is presented or the direction in which the arrow is pointing. The side and the direction are sometimes congruent (e.g., right-facing arrow presented on the right side), and sometimes incongruent (e.g., right-facing arrow presented on the left side). Among the assessed blocks, there are two single-task blocks (the side of the screen only and the direction of the arrow only), and one mixed block in which the two tasks switch randomly. The order of the blocks is fixed across participants. The typically assessed measures are: *mean correct reaction time* (RT; the overall speed of responding correctly, with means averaged across both congruent and incongruent trials—also known as global RT); *congruence cost*, calculated as the difference between RT to congruent trials and to incongruent trials (also known as interference score); *global switch cost*, the difference in processing time observed in single-task blocks (without the task switching) and nonwitch trials in a mixed block (also known as mixing cost); and finally, *local switch cost,* the difference between the RT observed in switch trials of the mixed block and in nonswitch trials of the mixed block (also known as switching cost; Kiesel et al., 2010; Koch et al., 2010; Monsell, 2003; Wiseheart et al., 2016). In addition to these typically assessed measures, we also derived the Sequential Congruency Effect (SCE; also known as the Gratton Effect), recently advocated as the potential candidate measure for comparing monolingual and bilingual speakers (Bialystok & Grundy, 2018; Goldsmith & Morton, 2018; Grundy et al., 2017). SCE was originally reported in research on EF as the asymmetry on congruency effect depending on the congruency within the previous trial. Namely, congruency effect (the advantage of congruent over incongruent trials) tends to be larger for the trials which were preceded by the congruent as compared with the trials which were preceded by the incongruent trials.

## Results

Detailed descriptive data are provided in the Supplementary Materials, and here we start each section by presenting them graphically. We then report the performance of our three groups of participants, Serbian monolinguals, Hungarian–Serbian bilinguals, and Slovak–Serbian bilinguals, task by task. We start by applying the linear mixed-effect regression models on raw responses, with random effects of participants and items. Multiple investigations have favored this approach over the traditional F1 and F2 analysis of variance (Baayen et al., 2008), particularly when analyzing bivariate responses, such as accuracy data (Jaeger, 2008; Popović Stijačić et al., 2018). The additional advantage of our approach is that we used multiple repeated measures as a single variable, keeping the information on the participant who provided the data for that measure. This way, instead of conducting multiple comparisons (i.e., comparing monolinguals and bilinguals on multitude of measures), we tested for the effects of meaningful structures behind the data (e.g., problem complexity, delay duration). For example, instead of comparing monolingual and bilingual participants for percent of correct responses for different set sizes separately, we tested for the effect of bilingualism and set size on response accuracy, thus gaining a more detailed insight. Within each analysis, the random structure was initially determined by following the recommendations of Barr et al. (2013) to include the maximum structure justified by the design. However, in order to prevent the overparameterization, the final random structure of the model was determined by comparing the nested models using the likelihood ratio test and by using the RePsychling package (Bates et al., 2015a, b; Matuschek et al., 2017). In addition to the linear mixed-effect regression, we also applied between-group comparisons by using nonparametric tests on aggregated data. This was undertaken for the comparison of monolingual and bilingual participants on the SWM task measure of Strategy, which is only available at the level of the aggregated data.

For the purposes of mixed-effect regression, we first adapted the detailed CANTAB outputs by transforming them to a long-data format suitable for this type of analysis. In the analyses which relied on reaction time, this variable was log-transformed prior to analysis (as suggested by Baayen & Milin, 2010). The analyses were run in R statistical software (R version 4.0.5; R Core Team, 2021), by using lme4 package (Bates et al., 2015a, b), ggplot2 (Wickham, 2016), sjPlot (Lüdecke, 2021), and lmertest (Kuznetsova et al., 2017). To avoid the influence of outliers, we refitted each model by excluding data points with residuals outside the range of ±2.5 units of standardized score. None of the refitted models differed in structure from their original variants. We reported the refitted models. For each test we analyzed only the assessed tasks (i.e., we excluded all data that were collected during practice or demo sessions). We did so by carefully following the instructions provided by CANTAB manuals.

### Delayed Matching to Sample (DMS)

**Fig. 1:**
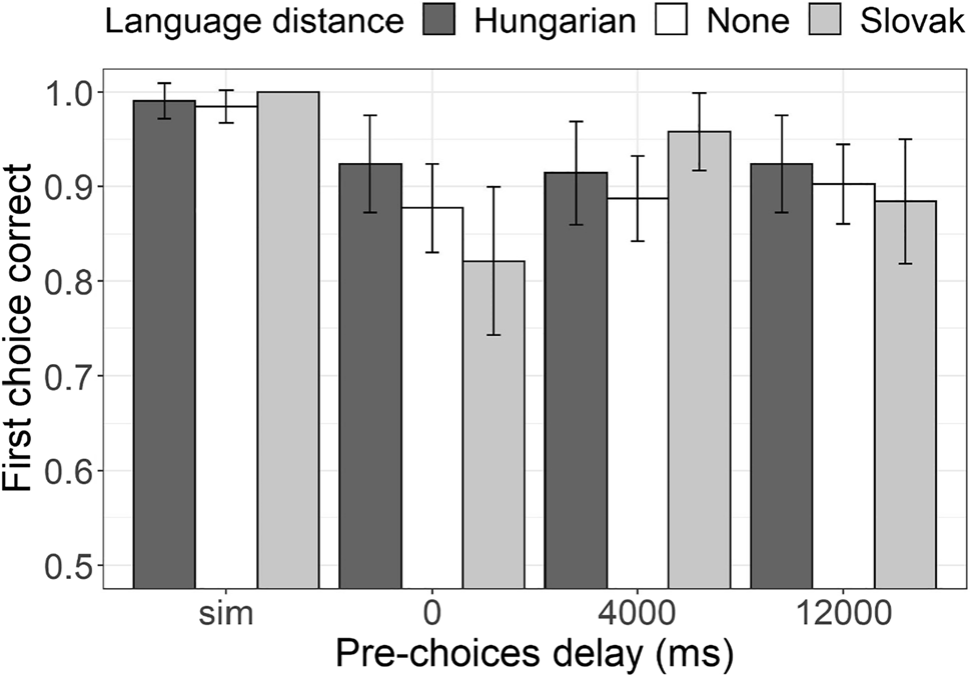
Proportion of correct first choice in DMS task of Hungarian–Serbian bilingual speakers (dark-gray bars), Slovak–Serbian bilingual speakers (light-gray bars), and monolingual Serbian speakers (white bars) across different delay conditions

The proportion of correct first choice responses is presented in Fig. 1. The data from the assessed trials were analyzed in a generalized linear mixed-effect regression in which language distance (*Hungarian*: large typological distance between Hungarian and Serbian, *Slovak*: small typological distance between Slovak and *none*: Serbian only) and prechoices delay (simultaneous, 0 ms, 4,000 ms, 12,000 ms) were included as the predictors of the first choice accuracy (1–accurate, 0–inaccurate). As presented in Table 2 (and Fig. 1), the analysis revealed only an effect of pre-choices delay: although highly accurate overall, our participants were less accurate in delayed conditions as compared with the simultaneous presentation. However, there were no differences among Serbian monolingual, Hungarian–Serbian bilingual, and Slovak–Serbian bilingual participants, and no interaction between language distance and pre-choices delay.

**Table 2:** The coefficients from the generalized linear mixed-effects model fitted to accuracy of the first choice (1–accurate; 0–inaccurate) in DMS task

	Accuracy of the first choice				
Predictors	*Odds Ratios*	*SE*	*CI*	*Statistic z*	*p*
Intercept (Language distance [Hungarian], Prechoices delay [Simultaneous])	115.99	121.33	14.93, 901.23	4.54	<.001
Language distance [None]	0.65	0.79	0.06, 6.96	−0.36	.721
Language distance [Slovak]	989837.34	1059311373.26	0.00, Inf	0.01	.990
Prechoices delay [0]	0.12	0.13	0.01, 1.02	−1.95	.052
Prechoices delay [12,000]	0.12	0.13	0.01, 1.02	−1.94	.052
Pre-choices delay [4,000]	0.10	0.11	0.01, 0.88	−2.07	.038
Language distance [None] * Prechoices delay [0]	0.92	1.18	0.07, 11.29	−0.07	.947
Language distance [Slovak] * Prechoices delay [0]	0.00	0.00	0.00, Inf	−0.01	.989
Language distance [None] * Prechoices delay [12,000]	1.20	1.54	0.10, 14.90	0.14	.886
Language distance [Slovak] * Prechoices delay [12,000]	0.00	0.00	0.00, Inf	−0.01	0.989
Language distance [None] * Prechoices delay [4,000]	1.16	1.48	0.10, 14.10	0.11	0.909
Language distance [Slovak] * Prechoices delay [4,000]	0.00	0.00	0.00, Inf	−0.01	0.990
Random Effects					
σ^2^	3.29				
τ_00 SubjectID_	0.29				
ICC	0.08				
N _SubjectID_	79				
Observations	1,580				
Marginal *R*^2^ / Conditional *R*^2^	0.806 / 0.822				

### Paired Associates Learning (PAL)

**Fig. 2:**
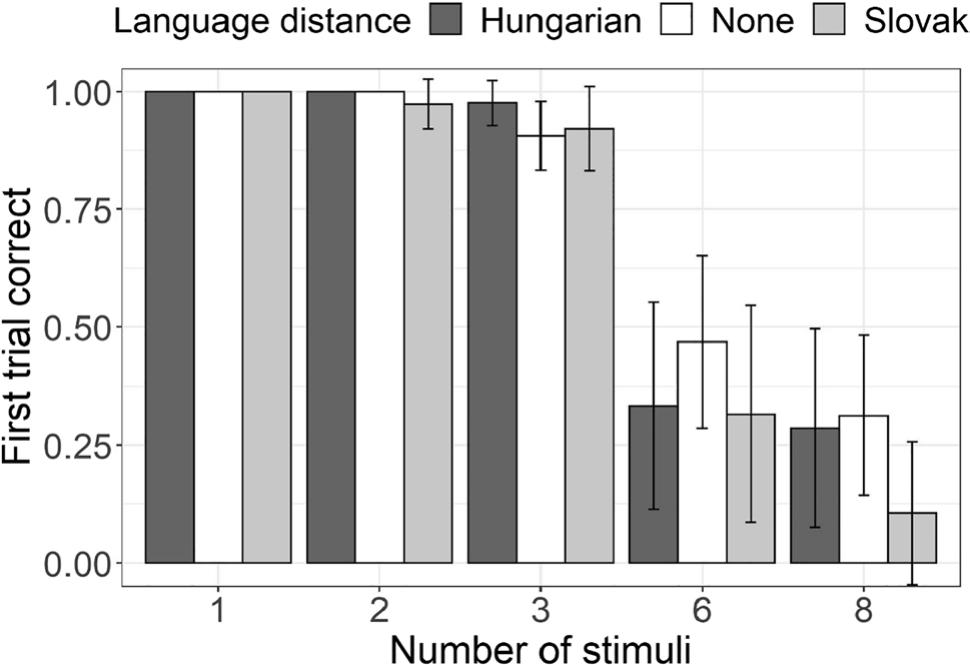
Proportion of the correct first trials in PAL task for Hungarian–Serbian bilingual speakers (dark-gray bars), Slovak–Serbian bilingual speakers (light-gray bars), and monolingual Serbian speakers (white bars) across trials with different number of presented stimuli

The proportion of the correct responses in the first attempt for the assessed trials in this task is presented in Fig. 2. The descriptive statistics showed that all participants were highly accurate when learning the association between visual identity and spatial location of up to three items. When the number of items was six or eight, our participants failed to learn this mapping regardless of their linguistic status. For the assessed trials we also performed generalized mixed-effects regression in which language distance (*Hungarian*, *Slovak*, *none*) and number of stimuli that was to be learned (1, 2, 3, 6, 8) were included as predictors (fixed effects) of accuracy in the first attempt (1–accurate, 0–inaccurate). The results presented in Table 3 show that there was a significant effect of the number of stimuli, which revealed that the increase in the complexity of the task was followed by a decrease in accuracy. This was true for all groups of participants (Hungarian–Serbian bilinguals, Slovak–Serbian bilinguals, and Serbian monolinguals). In addition to the null effect of language distance, no interaction was observed between the number of stimuli and language distance.

**Table 3:** The coefficients from the generalized linear mixed-effects model fitted to accuracy of the first attempt (1–accurate; 0–inaccurate) in PAL task

	Accuracy in the first attempt				
Predictors	*Odds Ratios*	*SE*	*CI*	*Statistic z*	*p*
Intercept (Language distance [Hungarian])	2091.69	3068.76	117.95 – 37094.49	5.21	<.001
Language distance [None]	0.25	0.34	0.02 – 3.53	−1.02	.307
Language distance [Slovak]	0.38	0.56	0.02 – 6.82	−0.66	.509
Number of stimuli	0.30	0.07	0.19 – 0.48	−4.97	<.001
Language distance [None] * Number of stimuli	1.27	0.27	0.84 – 1.92	1.13	.258
Language distance [Slovak] * Number of stimuli	1.03	0.25	0.64 – 1.66	0.13	.893
Random Effects					
σ^2^	3.29				
τ_00 SubjectID_	0.46				
τ_00 problem_number_	0.45				
ICC	0.22				
N _SubjectID_	72				
N _problem_number_	8				
Observations	576				
Marginal R^2^ / Conditional R^2^	0.615 / 0.698				

### Spatial Working Memory (SWM)

**Fig. 3:**
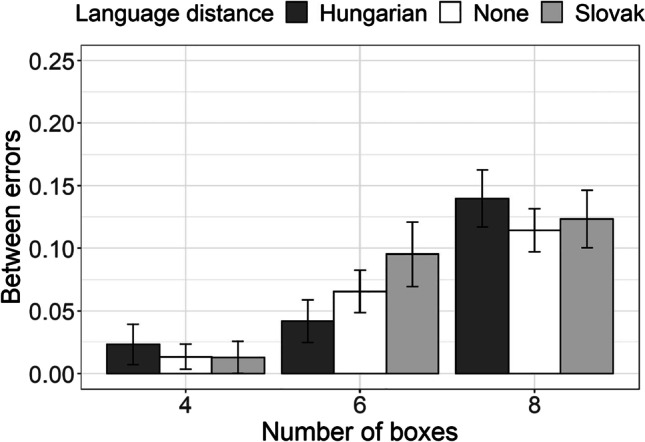
Proportion of between errors (revisits to already cleared boxes) in SWM task for the group of Hungarian–Serbian bilingual speakers (dark-gray bars), Slovak–Serbian bilingual speakers (light-gray bars), and monolingual Serbian speakers (white bars) for the tasks with four, six, and eight boxes

The first measure assessed in the SWM task was *the number of between errors*, which is the number of errors made by revisiting the boxes that had been cleared previously (Fig. 3). The results of the generalized mixed-effect regression fitted to accuracy of tapping on the box that had not been cleared previously revealed no difference between Hungarian–Serbian bilinguals, Slovak–Serbian bilinguals, and Serbian monolinguals (Table 4). All participants were more likely to make the between error (i.e., to tap the box that they had previously emptied) in problems which included eight boxes, as compared with problems including four boxes. However, this effect was identical for all groups, as no interaction between language distance and number of boxes was observed.

**Table 4:** The coefficients from the generalized linear mixed-effects model fitted to accuracy of the “tap” on the box, accurate tapping indicating the tap on the box that had not been emptied previously (1–erroneous tap on the already cleared box; 0–accurate tap on the box that has not been emptied previously) in the SWM task

	Between errors				
Predictors	*Odds Ratios*	*SE*	*CI*	*Statistic z*	*p*
Intercept (Language distance [Hungarian], Number of boxes [4])	0.00	0.00	0.00, 0.02	−5.21	<.001
Language distance [None]	0.55	0.63	0.06, 5.23	−0.52	.603
Language distance [Slovak]	0.67	0.85	0.05, 8.20	−0.32	.752
Number of boxes [6]	6.63	8.53	0.53, 82.50	1.47	.141
Number of boxes [8]	37.28	46.24	3.28, 423.92	2.92	.004
Language distance [None] * Number of boxes [6]	3.09	3.80	0.28, 34.29	0.92	.357
Language distance [Slovak] * Number of boxes [6]	5.36	7.28	0.37, 76.73	1.24	.216
Language distance [None] * Number of boxes [8]	1.46	1.77	0.14, 15.63	0.31	.754
Language distance [Slovak] * Number of boxes [8]	1.33	1.79	0.09, 18.69	0.21	.833
Random Effects					
σ^2^	3.29				
τ_00 subject_id_	6.14				
τ_11 subject_id.as.factor(Number of boxes)6_	6.67				
τ_11 subject_id.as.factor(Number of boxes)8_	7.36				
ρ_01_	−0.79				
	−0.83				
ICC	0.49				
N _subject_id_	72				
Observations	6,014				
Marginal *R*^2^ / Conditional *R*^2^	0.252 / 0.619				

The second measure assessed was *strategy* (i.e., the number of different boxes used by the participant to start the search). The participant who used fewer boxes when initializing the search was considered more successful in approaching the task. No significant difference between the strategy scores of monolinguals and bilinguals was observed: neither for Hungarian–Serbian bilinguals, *U*(21, 32) = 324.500, *Z* = .202, *p* = .841 (RankSum_HungarianBilingual_ = 578.500; RankSum_Monolingual_ = 825.500), nor for Slovak–Serbian bilinguals, *U*(19, 32) = 247.500, *Z* = 1.091, *p* = .272 (RankSum_SlovakBilingual_ = 550.500; RankSum_Monolingual_ = 775.500). Similarly, no such difference was found for the two bilingual groups either: *U*(19, 21) = 169.000, *Z* = .813, *p* = .416 (RankSum_Slovak_=420.000; RankSum_Hungarian_=400.000).

### Stockings of Cambridge (SOC)

#### Number of problems solved in a minimum number of moves

**Fig. 4:**
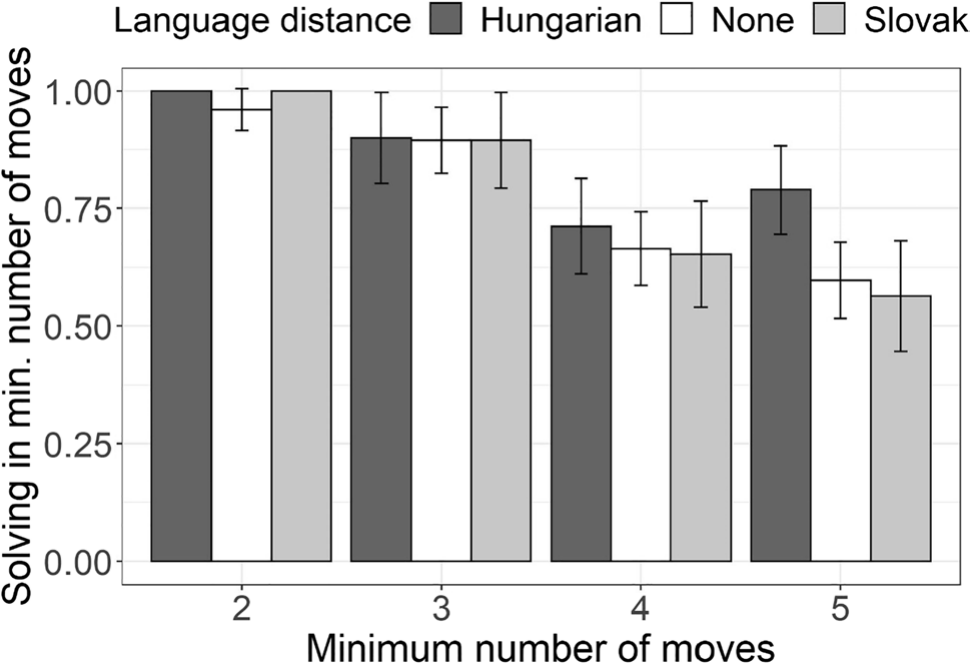
Proportion of problems solved in minimal number of moves by Hungarian–Serbian bilingual speakers (dark-gray bars), Slovak–Serbian bilingual speakers (light-gray bars), and monolingual Serbian speakers (white bars) for four categories of problems of varying complexity (x-axis: problems solved in minimally 2, 3, 4, and 5 moves)

Figure 4 presents the proportion of problems which were solved by applying the minimum number of moves for the two groups and across the four categories of problems (the rising number of minimum moves needed to solve the problem indicates the rising complexity of the problem in question). The more complex problems (i.e., problems that required more moves in order to be solved were less likely to be solved in a minimum number of moves). This seemed equally difficult for Serbian monolingual, Hungarian–Serbian bilingual, and Slovak–Serbian bilingual participants.

The results of the generalized linear mixed effect regression with language distance number of minimum moves (required to solve the problem) as predictors, and accuracy of solving the problem in minimum number of moves as the dependent variable (1–solved in minimum number of moves; 0–was not solved in minimum number of moves), and participants and problems as random effects, are shown in Table 5. The effect of the number of minimum moves was significant (i.e., the greater the number of moves which were minimally required to solve the problem, the smaller the probability that the problem was actually going to be solved in a minimum number of moves). However, there was no effect of language distance and no interaction between language distance and the number of moves. Therefore, the detrimental effect of the increase in problem complexity was the same for Serbian monolinguals, Hungarian–Serbian bilinguals, and Slovak–Serbian bilinguals (although there was a marginally significant tendency of Hungarian–Serbian bilinguals to be more successful than the remaining two groups as the complexity of problems increased—see second language by minimal number of moves interaction in Table 5).

**Table 5:** The coefficients from the generalized linear mixed-effects model fitted to accuracy in solving the problem in minimum number of moves (1–accurate; 0–inaccurate) in the SOC task

	Solving in minimal number of moves				
Predictors	*Odds Ratios*	*SE*	*CI*	*Statistic z*	*p*
Intercept (Language distance [Hungarian])	840.24	1767.81	13.60, 51912.77	3.20	0.001
Language distance [None]	7.92	11.33	0.48, 130.71	1.45	0.148
Language distance [Slovak]	7.33	11.99	0.30, 181.00	1.22	0.223
Minimal number of moves	0.31	0.15	0.12, 0.81	−2.39	0.017
Language distance [None] * Minimal number of moves	0.54	0.18	0.29, 1.03	−1.87	0.061
Language distance [Slovak] * Minimal number of moves	0.54	0.20	0.26, 1.11	−1.68	0.094
Random Effects					
σ^2^	3.29				
τ_00 subject_id_	0.25				
τ_00 problem_number_	1.37				
ICC	0.33				
N _subject_id_	79				
N _problem_number_	12				
Observations	893				
Marginal *R*^2^ / Conditional *R*^2^	0.389 / 0.591				

#### Initial thinking time

**Fig. 5:**
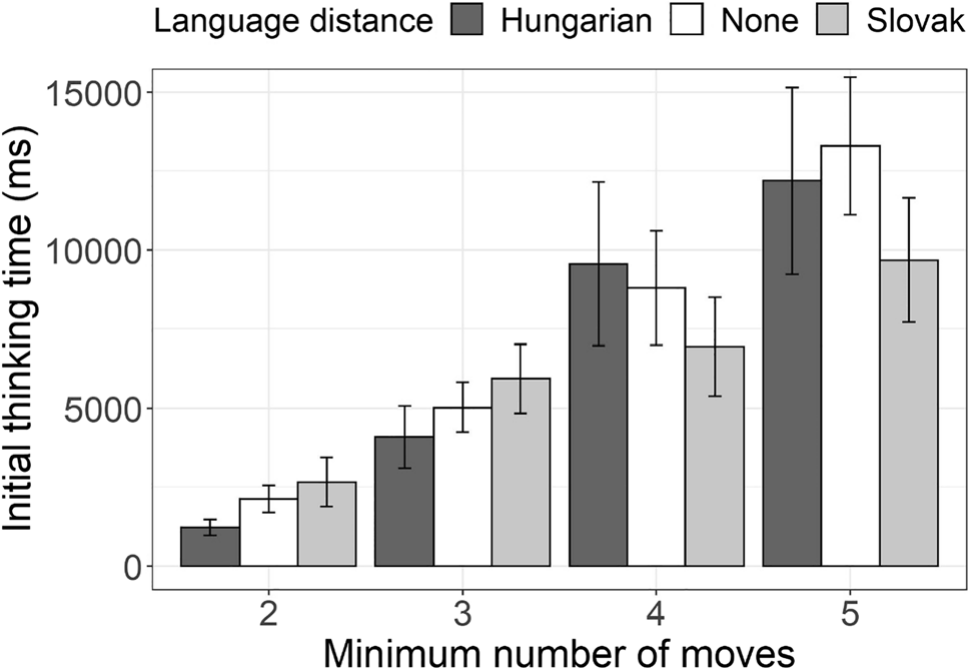
Initial thinking time (time spent in preparing prior to the first move) in SOC task for the Hungarian–Serbian bilingual speakers (dark-gray bars), Slovak–Serbian bilingual speakers (light-gray bars), and monolingual Serbian speakers (white bars) across the four levels of task complexity (four categories of tasks based on the number of minimal move required for solving the task)

Our results on the second measure of performance in SOC task, the time which the participant spent in preparing the moves prior to executing the first move, revealed that the problems which required greater number of moves to be solved (i.e., more complex problems) also consumed more preparation time (planning) prior to the execution of the first move (Fig. 5). However, unlike the number of problems solved in minimum moves, here, we observed a potential difference among the speaker groups.

Linear mixed-effect regression to log-transformed initial thinking time, with participants and SOC problems/tasks set as random effects, language distance and minimum number of moves (2, 3, 4, 5) as fixed effects revealed the expected significant effect of the number of moves (Table 6). However, it also revealed a significant effect of language distance, as well as language distance by minimal number of moves interaction. As observed in Fig. 5, Hungarian–Serbian bilinguals took less time to plan the execution of problems with two and three minimally required moves as compared with Serbian monolinguals and Slovak–Serbian bilinguals alike. However, for the problems of higher complexity this difference was not observed.

**Table 6:** The coefficients from the linear mixed-effects model fitted to initial thinking time in SOC task

	Initial thinking time				
Predictors	*Estimates*	*SE*	*CI*	*Statistic*	*p*
(Intercept)	6.84	0.16	6.52, 7.16	41.95	<.001
Second language [None]	0.49	0.19	0.12, 0.87	2.58	.010
Second language [Slovak]	0.80	0.22	0.36, 1.24	3.55	<.001
Minimal number of moves [3]	1.21	0.16	0.90, 1.52	7.65	<.001
Minimal number of moves [4]	1.71	0.14	1.44, 1.98	12.54	<.001
Minimal number of moves [5]	2.15	0.14	1.88, 2.42	15.66	<.001
Second language [None] * Minimal number of moves [3]	−0.28	0.17	−0.61, 0.06	−1.60	.109
Second language [Slovak] * Minimal number of moves [3]	−0.33	0.20	−0.72, 0.07	−1.64	.102
Second language [None] * Minimal number of moves [4]	−0.42	0.15	−0.71, −0.12	−2.78	.005
Second language [Slovak] * Minimal number of moves [4]	−0.82	0.18	−1.16, −0.47	−4.67	<.001
Second language [None] * Minimal number of moves [5]	−0.37	0.15	−0.67, −0.08	−2.49	.013
Second language [Slovak] * Minimal number of moves [5]	−0.91	0.18	−1.25, −0.56	−5.15	<.001
Random Effects					
σ^2^	0.38				
τ_00 subject_id_	0.30				
τ_00 problem_number_	0.01				
ICC	0.44				
N _subject_id_	79				
N _problem_number_	12				
Observations	882				
Marginal *R*^2^ / Conditional *R*^2^	0.353 / 0.640				

#### Initial thinking time for the problems that were solved in a nonminimum number of moves

**Fig. 6:**
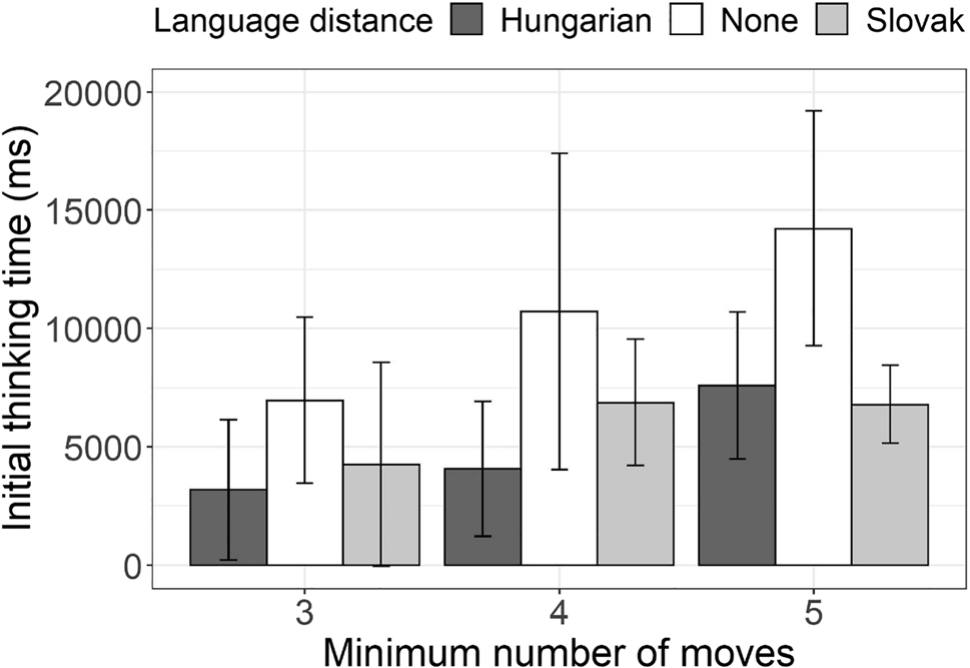
Initial thinking time for the SOC problems that were solved in nonminimal number of moves by Hungarian–Serbian bilingual speakers (dark-gray bars), Slovak–Serbian bilingual speakers (light-gray bars), and monolingual Serbian speakers (white bars) across the three categories of problem complexity with respect to minimal number of moves required for the problem to be solved (3, 4, and 5 moves)

Finally, in order to further explore potentials of this task, we reduced the dataset to those problems that were particularly hard for our participants (i.e., the problems that were successfully solved, but in a number of moves that was larger than the minimum number required). The category of problems that could be solved in two moves was excluded as none of these were solved in a nonminimum number of moves. The descriptive data presented in Fig. 6 suggest that these problems differentiate monolingual and bilingual participants.

To test the newly proposed variable, we selected the problems that were successfully solved in a nonminimum number of moves and performed a linear mixed-effect regression to initial thinking time. Participants and items were included as the random effects and language distance was included as the predictor. The results (Table 7) indeed revealed that the advantage of Hungarian–Serbian bilinguals over Slovak–Serbian bilinguals in taking less time to prepare for the execution of the moves needed to solve the simpler problems, showed a tendency of turning into advantage of both bilingual groups over the monolinguals. However, the number of data points which remained in this dataset is very small, hence this analysis should be treated as exploratory in nature.

**Table 7:** The coefficients from the generalized linear mixed-effects model fitted to initial thinking time for the problems that were solved in non-minimal number of moves in SOC task

	Initial thinking time				
Predictors	*Estimates*	*SE*	*CI*	*Statistic t*	*p*
Intercept (Language distance [Hungarian])	5.74	0.94	3.90, 7.58	6.11	<.001
Language distance [None]	1.89	1.07	−0.20, 3.98	1.77	.077
Language distance [Slovak]	2.83	1.18	0.52, 5.14	2.40	.016
Minimal number of moves	0.60	0.21	0.18, 1.01	2.79	.005
Language distance [None] * Minimal number of moves	−0.32	0.24	−0.78, 0.15	−1.33	.184
Language distance [Slovak] * Minimal number of moves	−0.59	0.26	−1.10,−0.07	−2.24	.025
Random Effects					
σ^2^	0.37				
τ_00 subject_id_	0.30				
τ_00 problem_number_	0.01				
ICC	0.46				
N _subject_id_	62				
N _problem_number_	10				
Observations	134				
Marginal *R*^2^ / Conditional *R*^2^	0.128 / 0.529				

**Fig. 7:**
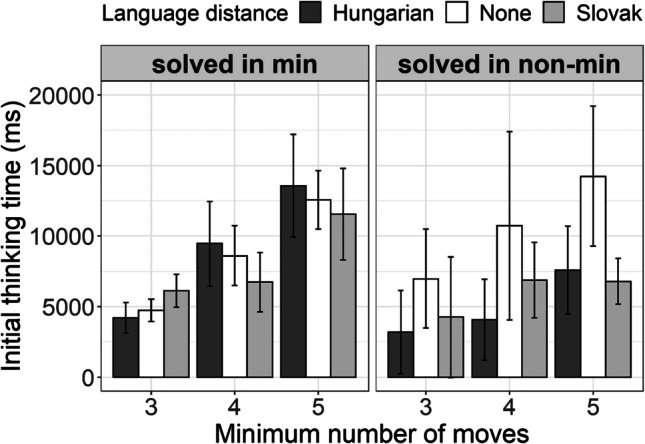
Initial thinking time for the problems that were solved in minimal number of moves (left-hand side) and those that were solved in non-minimal (i.e., greater than minimal) number of moves (right-hand side). Hungarian–Serbian bilingual speakers are represented by dark-gray bars, Slovak–Serbian bilingual speakers by light-gray bars, monolingual Serbian speakers by white bars, and x-axes represents three categories of problems with respect to the minimal number of moves required

Finally, Fig. 7 shows our comparison of initial thinking time for the two categories of successfully solved problems (approximately 90% of the total set—only around 10% of problems were not successfully solved): those that were solved in a minimum number of moves (left-hand side) and those that were solved in a nonminimum (i.e.. greater than minimal) number of moves (right-hand side). The difference between monolinguals and bilinguals is only present for the problems solved in a nonminimum number of moves.

### Attention Shifting Task (AST)

**Fig. 8:**
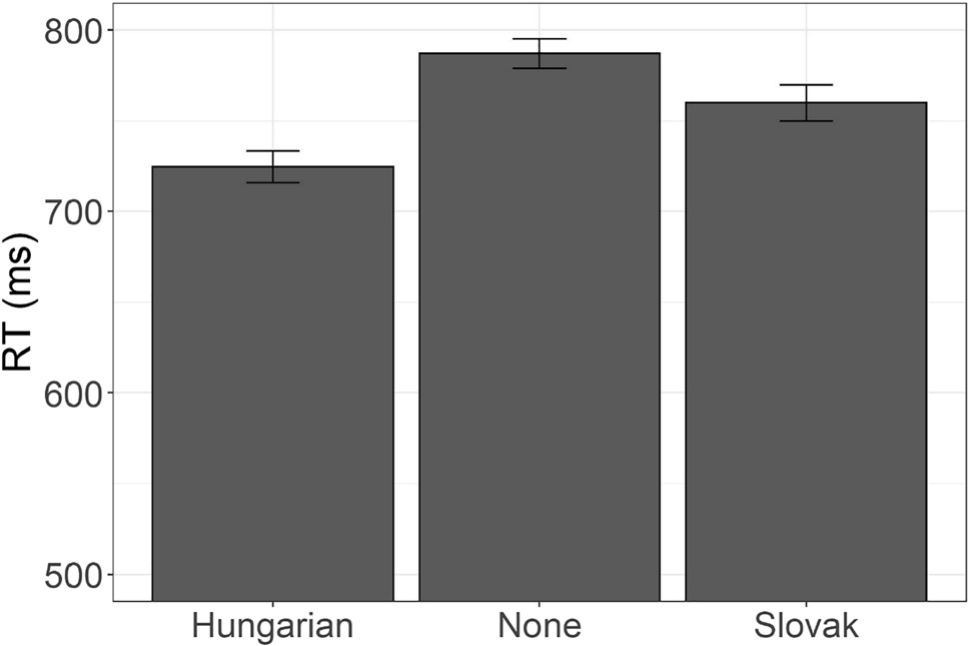
The average reaction time in AST for the Hungarian–Serbian bilingual speakers, Slovak–Serbian bilingual speakers, and monolingual Serbian speakers

Mean correct RT (i.e., the overall speed of the correct response) is presented in Fig. 8. A linear mixed-effect regression fitted to log-transformed RT in assessed correct trials revealed that Hungarian–Serbian bilingual participants were marginally faster as compared with Serbian monolinguals, but not compared with Slovak–Serbian bilinguals, which were in-between in processing speeds (Table 8).

**Table 8:** The coefficients from the linear mixed-effects model fitted to log-transformed reaction time in AST

	RT (ms)				
Predictors	*Estimates*	*SE*	*CI*	*Statistic t*	*p*
Intercept (Language distance [Hungarian])	6.52	0.04	6.46, 6.58	215.00	<.001
Language distance [None]	0.07	0.04	−0.01, 0.15	1.80	.072
Language distance [Slovak]	0.04	0.04	−0.04, 0.13	1.02	.308
Random Effects					
σ^2^	0.08				
τ_00 Subject_	0.02				
ICC	0.21				
N _Subject_	72				
Observations	10,859				
Marginal *R*^2^ / Conditional *R*^2^	0.009 / 0.204				

#### Congruence cost

**Fig. 9:**
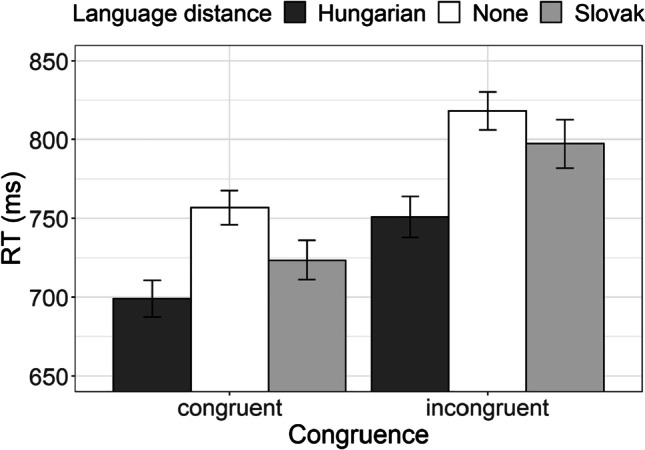
Reaction time to assessed correct trials in AST performance of the Hungarian–Serbian bilingual speakers (dark-gray bars), Slovak–Serbian bilingual speakers (light-gray bars), and monolingual Serbian speakers (white bars) in congruent and incongruent conditions

This measure is calculated as the difference between RT to congruent trials and to incongruent trials (Kiesel et al., 2010; Koch et al., 2010; Monsell, 2003; Wiseheart et al., 2016). We chose not to calculate the difference as the score (and compare it to zero), but to test for the effect of congruence (i.e., to compare RT in congruent and incongruent condition). Therefore, the effect of variable congruence would be equal to the existence of the congruence cost. In the same vein, the effect of bilingualism on congruence cost would correspond to the interaction between language distance and congruence. Figure 9 depicts average RT of the three groups of participants across congruent and incongruent trials.

We tested for the interaction between language distance and congruence by applying a linear mixed-effect regression to log-transformed RT to correct trials. We included participants as random effects, language distance and congruency (congruent, incongruent) as fixed effects. As presented in Table 9, we observed the expected advantage of congruent trials over incongruent trials (the congruence cost) and the reported marginally significant advantage of Hungarian–Serbian bilingual participants over monolingual participants. However, the interaction between the two variables was not significant, indicating that the difference between the RT in congruent and incongruent trials (i.e., the congruence cost) was identical for the three groups of participants. Thus, no effect of language distance on congruence cost was observed.

**Table 9:** The coefficients from the linear mixed-effects model fitted to AST reaction time as means of testing the congruence cost

	RT (ms)				
Predictors	*Estimates*	*SE*	*CI*	*Statistic*	*p*
Intercept (Language distance [Hungarian])	6.49	0.03	6.43, 6.55	211.48	<.001
Language distance[None]	0.07	0.04	−0.01, 0.14	1.69	.090
Language distance[Slovak]	0.04	0.04	−0.05, 0.13	0.88	.381
Congruence [Incongruent]	0.06	0.01	0.04, 0.08	6.06	<.001
Language distance [None] * Congruence [Incongruent]	0.01	0.01	−0.02, 0.03	0.56	.576
Language distance [Slovak] * Congruence [Incongruent]	0.01	0.01	−0.02, 0.04	0.87	.384
Random Effects					
σ^2^	0.08				
τ_00 Subject_	0.02				
ICC	0.20				
N _Subject_	72				
Observations	10,859				
Marginal *R*^2^ / Conditional *R*^2^	0.020 / 0.215				

**Fig. 10:**
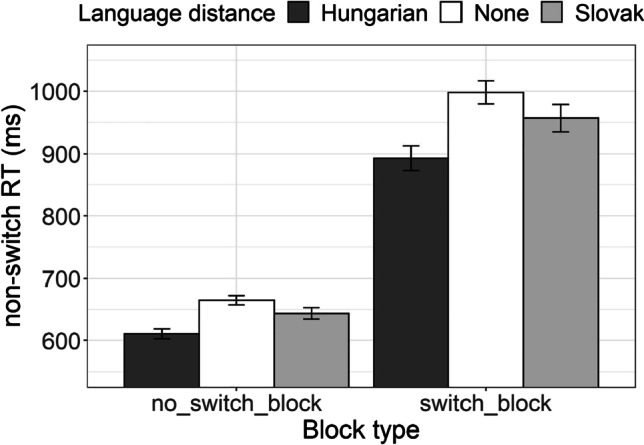
Average AST reaction time of Hungarian–Serbian bilingual speakers (dark-gray bars), Slovak–Serbian bilingual speakers (light-gray bars), and monolingual Serbian speakers (white bars) recorded for all trials in the nonswitch blocks and for nonswitch trials in the switch blocks

*Global switch cost* is usually calculated as the difference in RT recorded in blocks with a single task (no task switching) and nonswitch trials in a mixed block. As with the congruence cost, we chose to test for the effect of the block type (no-switch block, switch block) on RT instead of calculating the difference and comparing it to zero. This time, the effect of language distance on global switch cost would correspond to the interaction between language distance and block type. Figure 10 presents average RTs of the three groups of participants as observed in no switch blocks and in no-switch trials of the switch blocks.

In order to test for the interaction between language distance and block type we conducted a linear mixed-effect regression to log-transformed RT, with participant as random effects. The fixed effects were language distance and block type (no-switch block, switch block). As presented in Table 10, we observed the expected advantage of no-switch block over the switch block (the global switch cost) and the reported, now significant, advantage of Hungarian–Serbian bilingual participants over monolingual participants. However, although only marginally significant, the interaction between the two variables suggested that the observed advantage of Hungarian–Serbian bilinguals over Serbian monolinguals was more pronounced in the switch block than the nonswitch block. This suggested the existence of a marginal difference in global switch cost for our speaker groups and an advantage for the Hungarian–Serbian bilinguals.

**Table 10:** The coefficients from the linear mixed-effects model fitted to AST reaction time as means of testing the global switching cost

	RT (ms)				
Predictors	*Estimates*	*SE*	*CI*	*Statistic*	*p*
Intercept (Language distance [Hungarian])	6.38	0.03	6.32, 6.44	211.43	<.001
Language distance [None]	0.08	0.04	0.00, 0.16	2.05	.040
Language distance [Slovak]	0.05	0.04	−0.04, 0.14	1.14	.255
Block type [Switch block]	0.36	0.01	0.34, 0.38	37.46	<.001
Language distance [None] * Block type [Switch block]	0.02	0.01	−0.00, 0.05	1.80	.072
Language distance [Slovak] * Block type [Switch block]	0.02	0.01	−0.00, 0.05	1.72	.086
Random Effects					
σ^2^	0.05				
τ_00 Subject_	0.02				
ICC	0.27				
N _Subject_	72				
Observations	8,107				
Marginal *R*^2^ / Conditional *R*^2^	0.326 / 0.510				

**Fig. 11:**
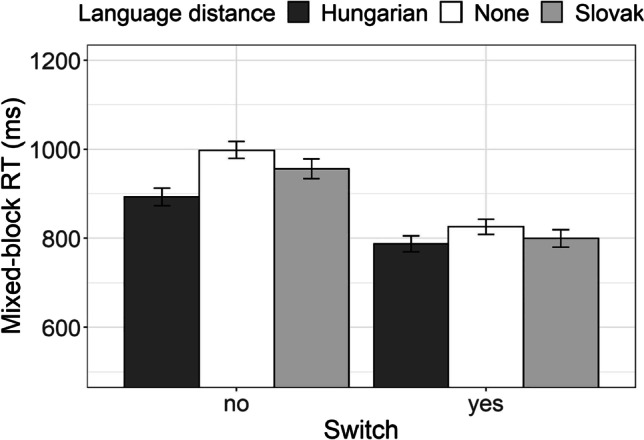
Average AST reaction time of Hungarian–Serbian bilingual speakers (dark-gray bars), Slovak–Serbian bilingual speakers (light-gray bars), and monolingual Serbian speakers (white bars) recorded for switch and nonswitch trials in the mixed block

*Local switch cost* is typically calculated as the difference between the RT recorded in switch trials of the mixed block and RT recorded in nonswitch trials of the mixed block. As with the previous two measures, we tested for the effect of the switch instead of calculating the difference in RT. The effect of language distance on local switch cost corresponds to the interaction between language distance and switch. Figure 11 presents average RTs of the three groups of participants which were recorded in no switch and switch trials of the mixed block.

We tested the interaction between language distance and switch by conducting a linear mixed-effect regression to log-transformed RT, with participants as random effects, as previously. The fixed effects were language distance and switch (no = no-switch trial, yes = switch trial). Table 11 presents the results of the analyses which again confirmed the reported advantage of Hungarian–Serbian bilingual participants over Serbian monolingual participants and the advantage of the nonswitch trials as compared with nonswitch trials (local switch effect). Crucially, the interaction between language distance and switch was significant: the difference between RT in no-switch trials and RT in switch trials of the mixed block (i.e., local switch cost) was smaller for Hungarian–Serbian bilinguals, indicating lower local switch cost for this group of participants, as compared with Serbian monolinguals and Slovak–Serbian bilinguals.

**Table 11:** The coefficients from the linear mixed-effects model fitted to AST reaction time as means of testing the local switching cost

	RT (ms)				
Predictors	*Estimates*	*SE*	*CI*	*Statistic*	*p*
Intercept (Language distance [Hungarian])	6.74	0.04	6.67, 6.81	187.82	<.001
Language distance [None]	0.11	0.05	0.02, 0.20	2.41	.016
Language distance [Slovak]	0.07	0.05	−0.03, 0.17	1.37	.171
Switch [yes]	−0.14	0.01	−0.16, −0.11	−10.45	<.001
Language distance [None] * Switch [yes]	−0.07	0.02	−0.11, −0.04	−4.28	<.001
Language distance [Slovak] * Switch [yes]	−0.06	0.02	−0.10, −0.02	−3.17	.002
Random Effects					
σ^2^	0.07				
τ_00 Subject_	0.03				
ICC	0.27				
N _Subject_	72				
Observations	5,449				
Marginal *R*^2^ / Conditional *R*^2^	0.094 / 0.338				

### Sequential Congruency Effect (SCE)

**Fig. 12:**
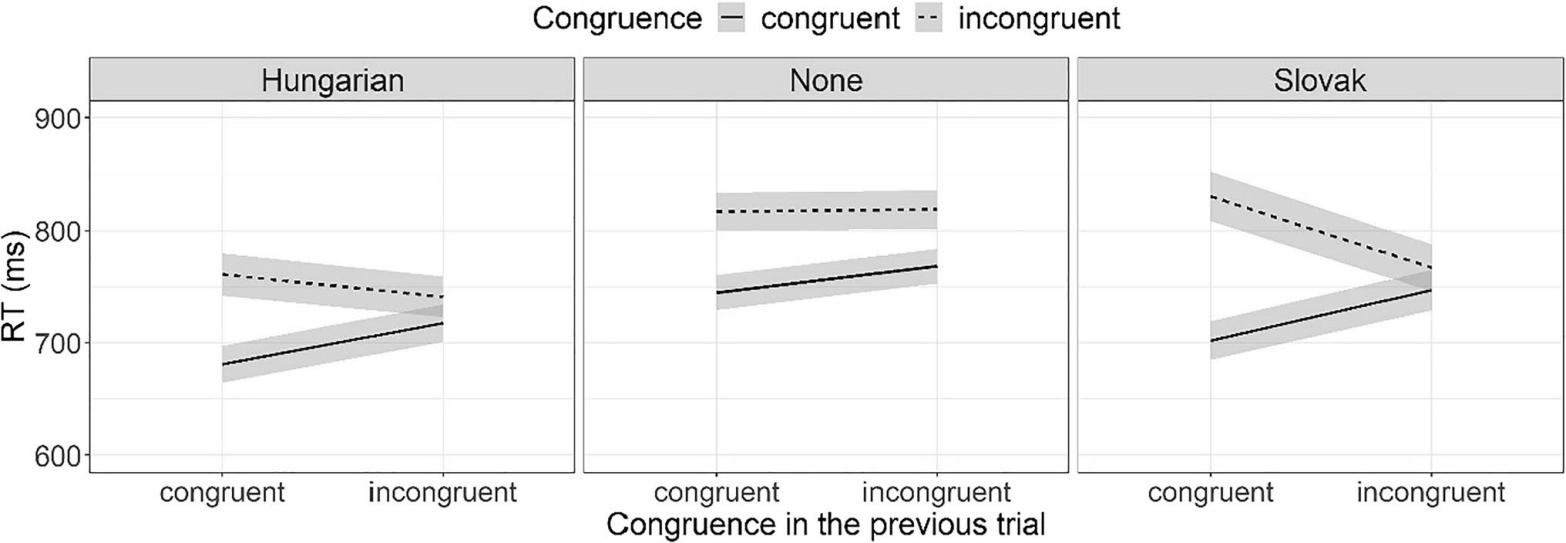
Average AST reaction time performance of Hungarian–Serbian bilingual speakers (dark-gray bars), Slovak–Serbian bilingual speakers (light-gray bars), and monolingual Serbian speakers (white bars) across different blocks for congruent and incongruent trials following previously congruent and incongruent trials

This effect manifests as the asymmetry in the congruency effect for trials preceded by congruent and incongruent trials. In other words, the finding of SCE indicates that the congruency effect (i.e., the advantage of congruent over incongruent trials) is larger following congruent trials than following incongruent trials. Figure 12 brings a typical SCE setting (Congruence × Previous Congruence) for the three groups of participants. Typical signature of the SCE is the “>” sign resembling plot, with the larger gap corresponding to a larger congruency effect following congruent trials. In terms of experimental design, SCE corresponds to interaction between congruence in the current trial and congruence in the previous one. The effect of language distance on SCE would, hence, be indicated by triple interaction of language distance, congruency and previous congruency.

To test for the diagnostic triple interaction, a linear mixed-effect regression model was fitted to log-transformed RT, with participants as the random effects (Table 12). The fixed effects were switch (yes, no), language distance, congruence (congruent, incongruent), and congruence in the previous trial (congruent, incongruent). We observed SCE for all groups of participants (as also visible by the characteristic “>” sign-like signature plots in Fig. 12). However, although the gap tended to be larger for the two bilingual groups, the critical triple interaction (Bilingualism × Congruency × Previous Congruency) was not significant.

**Table 12:** The coefficients from the linear mixed-effects model fitted to AST reaction time as means of testing the serial congruency effect (SCE)

	RT (ms)				
Predictors	*Estimates*	*SE*	*CI*	*Statistic*	*p*
Intercept (Language distance [Hungarian], Congruence [Congruent])	6.48	0.03	6.42, 6.54	211.21	<.001
Language distance [None]	0.08	0.04	−0.00, 0.15	1.96	.050
Language distance [Slovak]	0.04	0.04	−0.05, 0.13	0.86	.392
Congruence [Incongruent]	0.10	0.01	0.07, 0.13	6.73	<.001
Previous congruence [Incongruent]	0.04	0.01	0.01, 0.07	2.97	.003
Language distance [None] * Congruence [Incongruent]	−0.00	0.02	−0.04, 0.03	−0.23	.821
Language distance [Slovak] * Congruence [Incongruent]	0.04	0.02	0.00, 0.09	2.03	.042
Language distance [Slovak] * Previous congruence [Incongruent]	−0.01	0.02	−0.04, 0.03	−0.33	.741
Language distance [Slovak] * Previous congruence [Incongruent]	0.00	0.02	−0.04, 0.05	0.18	.855
Congruence [Incongruent] * Previous congruence [Incongruent]	−0.07	0.02	−0.11, −0.03	−3.18	.001
Language distance [Slovak] * Congruence [Incongruent] * Previous congruence [Incongruent]	0.02	0.03	−0.03, 0.07	0.74	.459
Language distance [Slovak] * Congruence [Incongruent] * Previous congruence [Incongruent]	−0.05	0.03	−0.11, 0.01	−1.62	.106
Random Effects					
σ^2^	0.09				
τ_00 Subject_	0.02				
ICC	0.16				
N _Subject_	72				
Observations	10,920				
Marginal *R*^2^ / Conditional *R*^2^	0.026 / 0.186				

To summarize, we compared the performance of three groups of participants—Hungarian–Serbian bilinguals (where Hungarian and Serbian are considered typologically distant languages), Slovak–Serbian bilinguals (where Slovak and Serbian are typologically similar languages), and Serbian monolinguals, in two groups of tasks—those that rely dominantly on memory (DMS, PAL, partly SWM), and those that rely dominantly on executive functions (partly SWM, SOC, AST). The three groups of participants exhibited equal performance in DMS, PAL, and SWM (both on the storage-based measure and the measure of executive functions). However, in simpler SOC tasks (which can be solved in a minimum of two, or three moves) Hungarian–Serbian bilinguals spent less time preparing (exhibiting a shorter initial thinking time) as compared with Serbian monolinguals and Slovak–Serbian bilinguals. This difference was not observed in more difficult SOC tasks (which can be solved in a minimum of four or five moves). For these complex tasks, the difference between bilingual and monolingual participants (but not between the two bilingual groups) seemed to emerge only for those tasks which were solved using more than a minimally required number of moves (those where participants struggled before successfully reaching the solution). Finally, in the AST task, although there were no differences in congruence cost and global switching cost (mixing cost), Hungarian–Serbian bilinguals were faster overall and demonstrated reduced local switching cost as compared both to Serbian monolinguals, and Slovak–Serbian bilinguals.

In short, we observed no differences among the three groups of participants in tasks that rely on memory. On the other hand, in tasks that rely on EF, we observed an advantage for high-distance bilinguals (Hungarian–Serbian) over both monolinguals and low-distance bilinguals (Slovak–Serbian). Importantly, although this advantage was moderate, it was observed in tasks that include multiple executive functions (SOC: inhibition; AST: attention switching, inhibition).

## Discussion

The current study was the first to investigate memory and EF in monolingual and bilingual speakers of three underresearched languages/language combinations: Serbian, Slovak–Serbian and Hungarian–Serbian. Our aim was to test the widely held but increasingly debated claim that there exists a bilingual advantage in tasks assessing EF in young adult bilinguals who are argued to be at the peak of their cognitive functioning. More specifically, we wanted to establish whether there exists an effect of language combination on the EF skills in speakers of typologically different languages: Slovak and Serbian, two Slavic languages, are closely related, while Hungarian and Serbian are not.

Taking into account concerns raised in recent reviews (e.g., Grundy, 2020; Leivada et al., 2021; Paap, 2019), we controlled for a number of factors that plagued previous studies reporting bilingual advantage in the literature. Our participants included monolinguals and early balanced bilinguals, university students, matched on place of origin and immigration status, and close in age and level of education. Our bilingual participants were exposed to their home languages from birth, or before the age of 3, and were at least partly educated in both of their languages. They were speakers of Hungarian or Slovak, two minority languages in the province of Vojvodina, Serbia, of similar status to the country’s majority language, Serbian. The performance of our three groups of participants was compared on a series of computerized nonverbal tasks of memory and EF from the CANTAB battery of neuropsychological tests suitable for data that may include ceiling performance in highly functioning healthy populations (Cambridge Cognition, 2016).

In terms of the tasks assessing different aspects of visual, episodic, and spatial memory (DMS, PAL, SWM), our results revealed no difference in the performance of the Hungarian–Serbian bilinguals, Slovak–Serbian bilinguals, and monolingual speakers. While the literature on the memory differences in bilinguals and monolinguals is inconclusive (see the meta-analyses referred to in the Introduction; Grundy & Timmer, 2017; Monnier et al., 2021), our results are in line with Ratiu and Azuma (2015) and Anjomshoae et al. (2021), who report no difference between young adult monolinguals and bilinguals on the tasks tapping into memory capacities. Both these studies are similar to ours in terms of participant population, though the results of Anjomshoae et al. (2021) are more relevant as they concern visuo-spatial memory, tested with the aid of a Corsi-Block tapping task.

On tasks tapping into EF, we observed some advantages in performance of Hungarian–Serbian bilinguals over monolingual speakers and the Slovak–Serbian bilinguals. The advantage observed in SOC could be interpreted as the effect of superior planning abilities. However, in line with Miyake et al. (2000) and Carder et al. (2004), we consider SOC to be tapping into inhibition: in order to solve the task, participants need to successfully inhibit inappropriate moves and select the appropriate ones. We shall espouse this interpretation of the data on SOC. On the AST task, we observed the advantage of Hungarian–Serbian bilinguals over Slovak–Serbian bilinguals and the monolingual speakers in global RT. Such advantage has been taken to indicate enhanced monitoring abilities (though see Paap, 2019, for arguments against such interpretations). Furthermore, although we did not observe neither congruence cost nor global switching cost, we did observe reduced mixing cost for Hungarian–Serbian bilinguals. These results are in line with studies most similar to ours in terms of homogeneity of participants: Hernández et al. (2013), and Branzi et al. (2018) also report an absence of global and switch cost effects for their samples of balanced Catalan–Spanish bilinguals. The last measure derived from AST, the Sequential Congruency Effect (SCE) revealed a (nonsignificant) tendency towards a larger SCE in our bilingual participants. Interestingly, Grundy et al. (2017) report a smaller SCE with bilinguals, interpreted as an indicator of their better disengagement of attention (but see the discussion in Paap, 2019, for an argument that a smaller or SCE need not be interpreted as an advantage). Note that Grundy et al. (2017) report no group differences in the reaction times on congruent and incongruent stimuli in their two experiments, in contrast to our results: our bilinguals were clearly faster than monolinguals on both the congruent and incongruent stimuli. Broadly, our results fit with traditional proposals (e.g., Green, 1998) that the observed patterns indicate a bilingual advantage in learning and cognitive control.

Overall, the pattern revealed in our study is the advantage of Hungarian–Serbian bilinguals over both Serbian monolinguals and Slovak–Serbian bilinguals on tasks tapping into EF. Whereas the advantage of bilingual speakers over monolinguals has been frequently addressed, divergence in the performance of bilinguals speaking different language combinations is seldom explored. We turn to this point below.

### The role of language typology

Why do Hungarian bilinguals outperform the Slovak bilinguals? Studies have noted the importance of bilingual experience when assessing bilingual advantage: age of language acquisition, language proficiency, language use, in addition to factors such as education and immigration status. Our two groups of bilingual participants did not differ on these variables; thus, this cannot be the explanation for the advantage of the Hungarian group, in contrast to predictions made by some literature (e.g., de Bruin, 2019). As established per our language background questionnaire, our bilinguals acquired both of their languages before the age of three; they were, on average, equally proficient in both their L1 and L2, and used both their languages equally frequently in a variety of contexts.

One variable emphasized by de Bruin (2019) for which we did not control is the interactional context of conversational exchanges. It has been argued that language-control abilities, and therefore cognitive-control abilities, are better in bilinguals who use both their languages in a dual-language context (i.e., both at work and at home, where they have to constantly monitor which of their languages is more appropriate to use; Green & Abutalebi, 2013). For bilinguals whose language interaction is restricted to a single-language context (i.e., one language is used at work and the other one at home), the demands of monitoring and inhibiting the appropriate language may not be so high. Recall from our review of the literature that the dual-language context was the key factor responsible for the better performance of bilinguals in Khodos et al. (2021). On a colour–shape switching task (based on Miyake et al., 2004), dual language use context was predictive of both mixing and switching costs, while earlier onset age of L2 and closer typological similarity of the language pairs spoken by participants were found to be predictive of mixing costs only. Their result of a typological similarity being associated with better performance on EF tasks are in direct contrast to ours: In our study, speakers of typologically unrelated languages outperformed the speakers of typologically similar languages. The explanation for the difference between their results and those reported here most likely involves the differences in the characteristics of bilinguals employed in the two studies. Recall that the sample in Khodos et al. was heterogeneous in a number of ways: their participants were late bilinguals who actively started using English only in adulthood, with differing levels of proficiency, and came from different cultural backgrounds, having immigrated to Australia as adults. They spoke a range of languages from widely different language families—Indo-European, Sino-Tibetan, Altaic, Benue-Congo—where language similarity was poorly defined. Future studies need to take into account potentially relevant factors such as proficiency and age of acquisition of L2 in order to establish whether these might interact with language similarity.

In contrast, our participant groups were highly homogenous in their demographic characteristics and their bilingual experience.^7^ Though our language background questionnaire did not probe into the use of languages in dual versus single switching contexts, it is likely that our two groups of bilingual participants did not differ in these respects*.* There are no reports of which context is more common in the Slovak communities in Vojvodina, but it has been reported that Hungarian–Serbian bilinguals use Hungarian primarily at home (i.e., the single-switching context; Halupka-Rešetar & Kovács Rácz, 2020). Thus, the only variable on which our two bilingual groups definitively differed was the typological relatedness of their two languages: The bilingual speakers whose first language was Hungarian, the language typologically unrelated to Serbian, showed a better performance than the speakers of Slovak, the language typologically related to Serbian.

The Hungarian bilinguals’ better performance in our study seems to support the view that the skills required to maintain separation between typologically unrelated languages lead to more enhanced executive functions. An explanation we would like to entertain (we thank an anonymous reviewer for pointing us in this direction) relies on recent findings of Branzi et al. (2020), which, while not invoking language typology per se, can be used to argue that bilingual speakers of languages that are lexically, phonologically, and grammatically distant (e.g., Hungarian and Serbian) are required to exert *more* cognitive control than speakers of languages that are more similar at the level of lexicon, phonology and/or grammar (e.g., Slovak and Serbian).

The exertion of more cognitive control thus may lead to an advantage in executive function for bilingual speakers of languages that are typologically distant, such as our Hungarian–Serbian bilinguals, compared with our Slovak–Serbian bilinguals. Building on studies that report multilinguals to engage in the process of preparing for the production of their target language, even before knowing which words to say (e.g., Reverberi et al., 2015), Branzi et al. (2020) found that language preparation may affect word retrieval differently for cognate versus noncognate words.^8^ The process of language preparation is known to bias activities in the mental lexicon of bilinguals and is likely to involve inhibition of the nontarget language: Branzi and colleagues suggest that this proactive language control modulates neural activation for noncognate, but not cognate representations. We speculate that similar processes are involved in bilinguals who speak typologically distant languages, compared with bilinguals who do not. In the typologically similar Slovak and Serbian, words often overlap in terms of phonology and meaning: the Serbian words “jabuka” (apple), “čitati” (to read), and “misliti” (to think) are very similar to Slovak “jablko,” “čítať,” “myslieť.” This is not the case for the typologically distant Serbian and Hungarian, where the relevant translations are phonologically distinct: “alma” (apple), “olvas” (to read), “gondol” (to think). Going back to Branzi et al.’s findings, we suggest that there exists a possibility that a stronger neural activation may also take place in the brains of speakers of typologically distant languages, as the mental lexicon of Hungarian–Serbian bilinguals contains fewer lexical overlaps (“fewer cognates”) than that of the Slovak–Serbian bilinguals. Future research should test these very tentative suggestions, relying on both behavioural methodology and neuroimaging, and evaluating a broader range of EFs.

### Limitations and future research

The restrictions of our inclusion criteria, whose aim was to make our participant groups as homogeneous as possible in terms of language spoken, language usage, educational background and immigration status, resulted in small sample size of our bilingual groups. The small sample size, as well as the lack of IQ matching, traditionally used in executive function research, must be considered when interpreting these findings. A further limitation concerns the issue of how “monolingual” our monolingual Serbian participants were: most young people growing up in Serbia will have been exposed to English as a foreign language at some level of their education. If the aim is to test young adults, at the peak of their functioning, they will by default speak a foreign language to some degree of proficiency. This reflects the reality of the modern world: purely monolingual young adult populations are exceptionally rare (Perquin et al., 2013). To control for this effect, we suggest that future studies also test participants’ proficiency in foreign languages taught at school. Note that even when not taking English exposure into account, it may be the case that the monolinguals in Vojvodina may not be the typical monolinguals found elsewhere. It has been suggested that the environments that provide us with “ideal” samples, homogenous in terms of bilingualism type, balanced language exposure and use (i.e., the border regions in Europe, such as Vojvodina, Catalonia, or Wales, that support strong inclusion language practices) are also environments that seem to breed a special category of monolinguals: according to Bice and Kroll (2019), growing up in multilingual communities makes one a better language learner overall.

### Conclusion

Our findings provide some support for studies reporting the enhanced EF in young adult bilinguals, the population for whom bilingualism effects have proven to be most unclear. The tasks from the CANTAB battery used here were shown to have the potential to assess even the most subtle differences in the performance of healthy young adults at the peak of their cognitive abilities. In one of the first studies to also control for the typological relatedness of languages spoken by the bilinguals, we observed that balanced bilinguals whose language combination includes typologically unrelated languages (Hungarian and Serbian) tend to perform better than the bilinguals whose language combination includes closely related languages (Slovak and Serbian), suggesting that the skills required to maintain separation between unrelated languages may lead to even more enhanced EF skills.

## Supplementary Information

ESM 1 (DOCX 44 kb)

## Footnotes

1 Our use of the term ‘bilingual’ only extends to individuals who speak one of the minority languages (Slovak or Hungarian) and the majority language (Serbian), and excludes any foreign languages taught at school, while the term ‘monolingual’ extends to individuals whose first and only home language is Serbian.

2 Ten bilinguals for whom we did not gather data on their bilingual background were subsequently excluded from the study, as was one bilingual for whom we had these data but who did not complete the full battery of tasks. To keep the sample as homogenous as possible in terms of age and education status, two further participants were excluded, one monolingual and one bilingual: They were not students at the University of Novi Sad and were significantly older (aged 40 and 44) than the general sample of student participants.

3 Due to time restrictions and the length of our task battery, we did not test our participants’ general IQ, which may have ensured even better matching between our bilinguals and monolinguals. However, as pointed out above, our groups were very close in terms of education, a variable often used as a proxy for IQ in the literature (see Deary & Johnson, 2010, for discussion). We return to this issue in the section pointing out the limitations of the current study.

4 Foreign language teaching in Serbia usually involves 2 or 3 hours teaching in classes of up to 30 students, from age 7, and is thus not considered to be equal to being exposed to home languages.

5 Responses to questions are each associated with a number on a scale; higher numbers indicate greater dominance in a language. Each module has between 4 and 6 questions, with questions worth between 0 and 6 points (Language Proficiency, Language Attitude), 0 and 10 points (Language Use) and 0 and 20 points (Language History) but each module receives equal weighting in the GLS (see Birdsong et al., 2012, for more detail).

6 CANTAB has been a popular choice in studies investigating cognitive changes associated with aging and dementia (e.g., Janssen et al., 2014), an important fact in view of research that bilingualism can stave off development of dementia symptoms (Bialystok, 2017). A computerized battery of tests that can be used online as well as in the lab may be particularly useful in times of epidemiological restrictions when access to research participants may be severely restricted (Backx et al., 2020).

7 The findings of Olguin et al. (2019) will not be discussed here: despite their population samples being similar to ours, where one group of bilinguals spoke a combination of typologically related languages (English–Dutch) and the other a combination of unrelated languages (English–Spanish), their methodology employing a dichotic listening task is not comparable to ours.

8 Cognates are words from distinct languages but with overlapping semantics, orthography and phonology: *tomato* in English versus *tomate* in Spanish. They contrast with noncognates, which are translation equivalents sharing only their meaning: *apple* in English versus *manzana* in Spanish (examples from Branzi et al., 2020).
