# Supplementary material for: The effect of bilingualism on executive functions when languages are similar: a comparison between Hungarian–Serbian and Slovak–Serbian young adult bilinguals
Source: Mem Cognit. 2022 Jul 29;51(3):561–81. doi: 10.3758/s13421-022-01345-8 (PMC9992248; doi:10.3758/s13421-022-01345-8)
Supplement: Supplementary file 1 — (DOCX 44 kb) [file 13421_2022_1345_MOESM1_ESM.docx]

**Supplementary material**

|  | **Monolingual participants** | | | | | | | |
| --- | --- | --- | --- | --- | --- | --- | --- | --- |
|  | N | \| Mean \| \| --- \| | \| Median \| \| --- \| | \| Minimum \| \| --- \| | \| Maximum \| \| --- \| | \| Lower Quartile \| \| --- \| | \| Upper Quartile \| \| --- \| | \| Std.Dev. \| \| --- \| |
| \| SOC Mean initial thinking time (2 moves) \| \| --- \| | 39 | 1911.73 | 1647.50 | 0.000 | 5800.00 | 980.000 | 2492.50 | 1320.453 |
| \| SOC Mean initial thinking time (3 moves) \| \| --- \| | 39 | 4933.56 | 4099.00 | 743.500 | 16077.00 | 3232.000 | 6458.00 | 2866.447 |
| \| SOC Mean initial thinking time (4 moves) \| \| --- \| | 39 | 8153.15 | 7038.25 | 992.500 | 27864.00 | 4124.750 | 10410.00 | 5604.688 |
| \| SOC Mean initial thinking time (5 moves) \| \| --- \| | 39 | 13391.39 | 10437.50 | 2873.250 | 41763.75 | 6410.500 | 18954.25 | 9296.229 |
| \| SOC Problems solved in minimum moves \| \| --- \| | 39 | 8.85 | 9.00 | 6.000 | 12.00 | 8.000 | 10.00 | 1.725 |
| \| DMS Percent correct (simultaneous) \| \| --- \| | 39 | 98.46 | 100.00 | 80.000 | 100.00 | 100.000 | 100.00 | 5.399 |
| \| DMS Percent correct (0 ms delay) \| \| --- \| | 39 | 89.23 | 100.00 | 40.000 | 100.00 | 80.000 | 100.00 | 14.397 |
| \| DMS Percent correct (4000 ms delay) \| \| --- \| | 39 | 90.26 | 100.00 | 60.000 | 100.00 | 80.000 | 100.00 | 13.667 |
| \| DMS Percent correct (12000 ms delay) \| \| --- \| | 39 | 92.31 | 100.00 | 60.000 | 100.00 | 80.000 | 100.00 | 11.801 |
| \| SWM Between errors (4 boxes) \| \| --- \| | 32 | 0.22 | 0.00 | 0.000 | 4.00 | 0.000 | 0.00 | 0.792 |
| \| SWM Between errors (6 boxes) \| \| --- \| | 32 | 1.69 | 0.50 | 0.000 | 13.00 | 0.000 | 2.00 | 2.923 |
| \| SWM Between errors (8 boxes) \| \| --- \| | 32 | 4.69 | 3.00 | 0.000 | 21.00 | 0.000 | 9.00 | 5.497 |
| \| SWM Strategy \| \| --- \| | 32 | 14.44 | 15.00 | 9.000 | 20.00 | 12.500 | 16.00 | 3.005 |
| \| AST Congruency cost (Mean, correct) \| \| --- \| | 32 | 63.80 | 60.84 | -37.225 | 189.20 | 39.530 | 82.58 | 48.988 |
| \| AST Congruency cost (Median, correct) \| \| --- \| | 32 | 66.67 | 57.25 | -21.000 | 200.00 | 28.000 | 103.75 | 51.501 |
| \| AST Congruency cost (Minimum, correct) \| \| --- \| | 32 | -10.78 | 3.00 | -95.000 | 43.00 | -44.000 | 16.50 | 38.244 |
| \| AST Percent correct trials \| \| --- \| | 32 | 98.44 | 98.75 | 95.625 | 100.00 | 97.500 | 99.38 | 1.219 |
| \| AST Switching cost (Mean, correct) \| \| --- \| | 32 | 245.99 | 223.99 | 54.241 | 697.25 | 174.594 | 310.23 | 129.557 |
| \| AST Switching cost (Median, correct) \| \| --- \| | 32 | 210.92 | 187.75 | 17.000 | 682.50 | 125.000 | 274.25 | 131.917 |
| \| AST Switching cost (Minimum, correct) \| \| --- \| | 32 | 53.91 | 40.50 | -100.000 | 206.00 | 2.500 | 120.00 | 76.462 |
| \| AST Switching cost (Maximum, correct) \| \| --- \| | 32 | 489.22 | 560.50 | -301.000 | 1116.00 | 134.000 | 766.00 | 387.835 |
| \| AST Congruency cost (Maximum, correct) \| \| --- \| | 32 | 25.88 | 5.00 | -700.000 | 779.00 | -122.500 | 197.00 | 299.765 |
| \| PAL First trial memory score \| \| --- \| | 32 | 22.59 | 23.50 | 17.000 | 26.00 | 20.500 | 24.00 | 2.650 |

|  | **Hungarian-Serbian bilingual participants** | | | | | | | |
| --- | --- | --- | --- | --- | --- | --- | --- | --- |
|  | \| N \| \| --- \| | \| Mean \| \| --- \| | \| Median \| \| --- \| | \| Minimum \| \| --- \| | \| Maximum \| \| --- \| | \| Lower Quartile \| \| --- \| | \| Upper Quartile \| \| --- \| | \| Std.Dev. \| \| --- \| |
| \| SOC Mean initial thinking time (2 moves) \| \| --- \| | 21 | 1173.62 | 1014.500 | 41.500 | 3071.50 | 692.500 | 1425.50 | 694.287 |
| \| SOC Mean initial thinking time (3 moves) \| \| --- \| | 21 | 4009.12 | 3175.500 | 794.500 | 9266.50 | 2388.500 | 5055.50 | 2337.732 |
| \| SOC Mean initial thinking time (4 moves) \| \| --- \| | 21 | 9294.80 | 7404.250 | 1128.250 | 29279.00 | 3240.750 | 9867.75 | 8261.249 |
| \| SOC Mean initial thinking time (5 moves) \| \| --- \| | 21 | 11897.62 | 9230.250 | 1203.750 | 37972.50 | 7048.000 | 14929.00 | 9510.093 |
| \| SOC Problems solved in minimum moves \| \| --- \| | 21 | 9.76 | 10.000 | 6.000 | 12.00 | 9.000 | 11.00 | 1.700 |
| \| DMS Percent correct (simultaneous) \| \| --- \| | 21 | 99.05 | 100.000 | 80.000 | 100.00 | 100.000 | 100.00 | 4.364 |
| \| DMS Percent correct (0 ms delay) \| \| --- \| | 21 | 92.38 | 100.000 | 80.000 | 100.00 | 80.000 | 100.00 | 9.952 |
| \| DMS Percent correct (4000 ms delay) \| \| --- \| | 21 | 91.43 | 100.000 | 60.000 | 100.00 | 80.000 | 100.00 | 11.952 |
| \| DMS Percent correct (12000 ms delay) \| \| --- \| | 21 | 92.38 | 100.000 | 60.000 | 100.00 | 80.000 | 100.00 | 11.792 |
| \| SWM Between errors (4 boxes) \| \| --- \| | 21 | 0.38 | 0.000 | 0.000 | 5.00 | 0.000 | 0.00 | 1.161 |
| \| SWM Between errors (6 boxes) \| \| --- \| | 21 | 1.05 | 0.000 | 0.000 | 8.00 | 0.000 | 1.00 | 2.156 |
| \| SWM Between errors (8 boxes) \| \| --- \| | 21 | 5.90 | 5.000 | 0.000 | 19.00 | 0.000 | 11.00 | 6.434 |
| \| SWM Strategy \| \| --- \| | 21 | 14.71 | 15.000 | 9.000 | 22.00 | 13.000 | 17.00 | 3.052 |
| \| AST Congruency cost (Mean, correct) \| \| --- \| | 21 | 50.44 | 53.220 | -0.870 | 89.54 | 38.578 | 67.32 | 27.098 |
| \| AST Congruency cost (Median, correct) \| \| --- \| | 21 | 52.95 | 57.500 | -19.500 | 120.00 | 31.000 | 66.50 | 31.554 |
| \| AST Congruency cost (Minimum, correct) \| \| --- \| | 21 | -1.29 | -6.000 | -47.000 | 64.00 | -30.000 | 22.00 | 34.145 |
| \| AST Percent correct trials \| \| --- \| | 21 | 98.75 | 98.750 | 96.250 | 100.00 | 98.750 | 99.38 | 1.046 |
| \| AST Switching cost (Mean, correct) \| \| --- \| | 21 | 228.52 | 212.483 | 77.177 | 405.48 | 132.532 | 339.90 | 109.467 |
| \| AST Switching cost (Median, correct) \| \| --- \| | 21 | 197.40 | 203.000 | 60.000 | 360.00 | 109.500 | 296.00 | 102.397 |
| \| AST Switching cost (Minimum, correct) \| \| --- \| | 21 | 82.95 | 59.000 | -25.000 | 196.00 | 50.000 | 133.00 | 54.911 |
| \| AST Switching cost (Maximum, correct) \| \| --- \| | 21 | 431.67 | 475.000 | -363.000 | 983.00 | 300.000 | 704.00 | 342.590 |
| \| AST Congruency cost (Maximum, correct) \| \| --- \| | 21 | 21.24 | 44.000 | -481.000 | 363.00 | -92.000 | 201.00 | 220.434 |
| \| PAL First trial memory score \| \| --- \| | 21 | 21.81 | 23.000 | 15.000 | 26.00 | 20.000 | 24.00 | 3.444 |

|  | **Slovak-Serbian bilingual participants** | | | | | | | |
| --- | --- | --- | --- | --- | --- | --- | --- | --- |
|  | \| N \| \| --- \| | \| Mean \| \| --- \| | \| Median \| \| --- \| | \| Minimum \| \| --- \| | \| Maximum \| \| --- \| | \| Lower Quartile \| \| --- \| | \| Upper Quartile \| \| --- \| | \| Std.Dev. \| \| --- \| |
| \| SOC Mean initial thinking time (2 moves) \| \| --- \| | 19 | 2587.868 | 2003.500 | 88.500 | 9391.50 | 935.000 | 3806.00 | 2196.657 |
| \| SOC Mean initial thinking time (3 moves) \| \| --- \| | 19 | 5922.316 | 5991.500 | 1510.000 | 10624.50 | 3302.500 | 7611.50 | 2533.013 |
| \| SOC Mean initial thinking time (4 moves) \| \| --- \| | 19 | 7119.303 | 6339.500 | 1403.750 | 20278.25 | 3659.750 | 9475.50 | 4601.546 |
| \| SOC Mean initial thinking time (5 moves) \| \| --- \| | 19 | 9627.645 | 7731.250 | 1363.750 | 24625.75 | 5665.250 | 12515.25 | 5936.331 |
| \| SOC Problems solved in minimum moves \| \| --- \| | 19 | 8.632 | 9.000 | 5.000 | 11.00 | 8.000 | 10.00 | 1.606 |
| \| DMS Percent correct (simultaneous) \| \| --- \| | 19 | 100.000 | 100.000 | 100.000 | 100.00 | 100.000 | 100.00 | 0.000 |
| \| DMS Percent correct (0 ms delay) \| \| --- \| | 19 | 82.105 | 80.000 | 60.000 | 100.00 | 60.000 | 100.00 | 17.505 |
| \| DMS Percent correct (4000 ms delay) \| \| --- \| | 19 | 95.789 | 100.000 | 80.000 | 100.00 | 100.000 | 100.00 | 8.377 |
| \| DMS Percent correct (12000 ms delay) \| \| --- \| | 19 | 88.421 | 100.000 | 40.000 | 100.00 | 80.000 | 100.00 | 18.032 |
| \| SWM Between errors (4 boxes) \| \| --- \| | 19 | 0.211 | 0.000 | 0.000 | 3.00 | 0.000 | 0.00 | 0.713 |
| \| SWM Between errors (6 boxes) \| \| --- \| | 19 | 2.526 | 1.000 | 0.000 | 9.00 | 0.000 | 5.00 | 3.044 |
| \| SWM Between errors (8 boxes) \| \| --- \| | 19 | 5.105 | 5.000 | 0.000 | 15.00 | 0.000 | 11.00 | 5.301 |
| \| SWM Strategy \| \| --- \| | 19 | 15.526 | 16.000 | 9.000 | 23.00 | 13.000 | 19.00 | 4.128 |
| \| AST Congruency cost (Mean, correct) \| \| --- \| | 19 | 75.771 | 72.096 | -9.714 | 215.04 | 30.644 | 108.57 | 57.416 |
| \| AST Congruency cost (Median, correct) \| \| --- \| | 19 | 62.105 | 57.000 | -36.000 | 203.00 | 26.000 | 76.00 | 55.145 |
| \| AST Congruency cost (Minimum, correct) \| \| --- \| | 19 | 12.158 | 9.000 | -140.000 | 105.00 | -3.000 | 43.00 | 53.802 |
| \| AST Percent correct trials \| \| --- \| | 19 | 96.678 | 98.125 | 74.375 | 100.00 | 96.875 | 99.38 | 5.863 |
| \| AST Switching cost (Mean, correct) \| \| --- \| | 19 | 231.125 | 215.584 | 88.225 | 400.05 | 172.594 | 297.87 | 82.774 |
| \| AST Switching cost (Median, correct) \| \| --- \| | 19 | 191.553 | 180.500 | 66.500 | 355.00 | 117.500 | 261.00 | 82.277 |
| \| AST Switching cost (Minimum, correct) \| \| --- \| | 19 | 63.316 | 41.000 | -43.000 | 306.00 | 9.000 | 117.00 | 84.737 |
| \| AST Switching cost (Maximum, correct) \| \| --- \| | 19 | 525.105 | 602.000 | -137.000 | 1120.00 | 331.000 | 789.00 | 347.410 |
| \| AST Congruency cost (Maximum, correct) \| \| --- \| | 19 | 200.737 | 206.000 | -461.000 | 992.00 | 72.000 | 343.00 | 359.909 |
| \| PAL First trial memory score \| \| --- \| | 19 | 20.842 | 22.000 | 12.000 | 25.00 | 20.000 | 23.00 | 3.387 |

| Variable | **Overall** | | | | | | | |
| --- | --- | --- | --- | --- | --- | --- | --- | --- |
|  | \| N \| \| --- \| | \| Mean \| \| --- \| | \| Median \| \| --- \| | \| Minimum \| \| --- \| | \| Maximum \| \| --- \| | \| Lower Quartile \| \| --- \| | \| Upper Quartile \| \| --- \| | \| Std.Dev. \| \| --- \| |
| \| SOC Mean initial thinking time (2 moves) \| \| --- \| | 79 | 1878.14 | 1559.000 | 0.000 | 9391.50 | 898.500 | 2393.50 | 1530.840 |
| \| SOC Mean initial thinking time (3 moves) \| \| --- \| | 79 | 4925.63 | 4471.000 | 743.500 | 16077.00 | 2900.000 | 6458.00 | 2711.644 |
| \| SOC Mean initial thinking time (4 moves) \| \| --- \| | 79 | 8207.98 | 6882.500 | 992.500 | 29279.00 | 3855.250 | 10354.00 | 6188.511 |
| \| SOC Mean initial thinking time (5 moves) \| \| --- \| | 79 | 12089.11 | 9949.000 | 1203.750 | 41763.75 | 6410.500 | 15237.75 | 8703.920 |
| \| SOC Problems solved in minimum moves \| \| --- \| | 79 | 9.04 | 9.000 | 5.000 | 12.00 | 8.000 | 10.00 | 1.728 |
| \| DMS Percent correct (simultaneous) \| \| --- \| | 79 | 98.99 | 100.000 | 80.000 | 100.00 | 100.000 | 100.00 | 4.413 |
| \| DMS Percent correct (0 ms delay) \| \| --- \| | 79 | 88.35 | 100.000 | 40.000 | 100.00 | 80.000 | 100.00 | 14.538 |
| \| DMS Percent correct (4000 ms delay) \| \| --- \| | 79 | 91.90 | 100.000 | 60.000 | 100.00 | 80.000 | 100.00 | 12.203 |
| \| DMS Percent correct (12000 ms delay) \| \| --- \| | 79 | 91.39 | 100.000 | 40.000 | 100.00 | 80.000 | 100.00 | 13.467 |
| \| SWM Between errors (4 boxes) \| \| --- \| | 72 | 0.26 | 0.000 | 0.000 | 5.00 | 0.000 | 0.00 | 0.888 |
| \| SWM Between errors (6 boxes) \| \| --- \| | 72 | 1.72 | 0.000 | 0.000 | 13.00 | 0.000 | 2.50 | 2.774 |
| \| SWM Between errors (8 boxes) \| \| --- \| | 72 | 5.15 | 3.000 | 0.000 | 21.00 | 0.000 | 11.00 | 5.678 |
| \| SWM Strategy \| \| --- \| | 72 | 14.81 | 15.000 | 9.000 | 23.00 | 13.000 | 17.00 | 3.330 |
| \| AST Congruency cost (Mean, correct) \| \| --- \| | 72 | 63.06 | 60.378 | -37.225 | 215.04 | 38.313 | 87.72 | 46.702 |
| \| AST Congruency cost (Median, correct) \| \| --- \| | 72 | 61.47 | 57.250 | -36.000 | 203.00 | 29.000 | 81.25 | 47.363 |
| \| AST Congruency cost (Minimum, correct) \| \| --- \| | 72 | -1.96 | 3.000 | -140.000 | 105.00 | -30.500 | 23.50 | 42.302 |
| \| AST Percent correct trials \| \| --- \| | 72 | 98.06 | 98.750 | 74.375 | 100.00 | 97.500 | 99.38 | 3.223 |
| \| AST Switching cost (Mean, correct) \| \| --- \| | 72 | 236.97 | 215.395 | 54.241 | 697.25 | 154.781 | 317.81 | 111.840 |
| \| AST Switching cost (Median, correct) \| \| --- \| | 72 | 201.87 | 187.750 | 17.000 | 682.50 | 113.250 | 275.00 | 111.082 |
| \| AST Switching cost (Minimum, correct) \| \| --- \| | 72 | 64.86 | 56.500 | -100.000 | 306.00 | 19.500 | 123.50 | 73.307 |
| \| AST Switching cost (Maximum, correct) \| \| --- \| | 72 | 481.90 | 538.500 | -363.000 | 1120.00 | 204.000 | 753.50 | 361.392 |
| \| AST Congruency cost (Maximum, correct) \| \| --- \| | 72 | 70.67 | 65.500 | -700.000 | 992.00 | -118.000 | 228.50 | 303.176 |
| \| PAL First trial memory score \| \| --- \| | 72 | 21.90 | 23.000 | 12.000 | 26.00 | 20.000 | 24.00 | 3.136 |
